# Loss of endothelial ZEB2 in mice attenuates steatosis early during metabolic dysfunction-associated steatotic liver disease

**DOI:** 10.1038/s41598-025-05881-6

**Published:** 2025-07-02

**Authors:** Wouter Dheedene, Stefaan Verhulst, Louise Demuynck, Bram Callewaert, Willeke de Haan, Stefan Vinckier, Jore Van Wauwe, Petra Vandervoort, Marleen Lox, Mathias Stroobants, Renaud Lavend’homme, Wilfred F. J. van IJcken, Elizabeth A. V. Jones, An Zwijsen, Marc Jacquemin, Leo A. van Grunsven, Kimberly Martinod, Danny Huylebroeck, Eskeatnaf Mulugeta, Aernout Luttun

**Affiliations:** 1 Department of Cardiovascular Sciences, Center for Molecular and Vascular Biology, Endothelial Cell Biology Unit, KU Leuven, Campus Gasthuisberg, Onderwijs & Navorsing 1, Herestraat 49, box 911, Leuven, 3000 Belgium; 2 Liver Cell Biology research group, Vrije Universiteit Brussel, Brussels, Belgium; 3 Department of Oncology, Laboratory of Angiogenesis and Vascular Metabolism, KU Leuven, Leuven, Belgium; 4 Laboratory of Angiogenesis and Vascular Metabolism, Center for Cancer Biology, VIB, Leuven, Belgium; 5 Center for Biomics-Genomics, Erasmus University Medical Center, Rotterdam, The Netherlands; 6 Department of Cell Biology, Erasmus University Medical Center, Rotterdam, The Netherlands; 7 CARIM, Maastricht University, Maastricht, The Netherlands; 8 Department of Development and Regeneration, KU Leuven, Leuven, Belgium; 9 Department of Internal Medicine, Erasmus University Medical Center, Rotterdam, The Netherlands

**Keywords:** Liver sinusoidal endothelial cells, Metabolic dysfunction-associated steatotic liver disease, Peroxisome Proliferator-Activated receptor alpha, Zinc-finger E-Box-binding Homeobox 2, Capillarization, Transcriptomics, Non-alcoholic fatty liver disease

## Abstract

Metabolic dysfunction-associated steatotic liver disease (MASLD), formerly known as non-alcoholic fatty liver disease, features liver sinusoidal endothelial cell (LSEC) alterations with ill-defined driving factors. Zinc-Finger E-Box-binding Homeobox (ZEB)2 in LSECs preserves their specialized features, prevents capillarization and protects against liver fibrosis. To investigate a potential protective role against steatosis, the initial MASLD stage, we fed EC-specific *Zeb2* knockout (*EC*^*Zeb2KO*^) mice a western-type diet (WD). In healthy and steatotic *wild-type* livers, *Zeb2* was ubiquitously and similarly expressed across blood-vascular EC types. LSEC RNA sequencing revealed ZEB2 deficiency-triggered expression changes greatly overlapping with those evoked by WD-feeding. Endothelial ZEB2-loss and WD-feeding interacted to boost capillarization and fat metabolism, shown by increased expression of continuous EC markers and peroxisome proliferator-activated receptor (PPAR)α signaling components, respectively. Altered communication among LSECs after combined endothelial ZEB2-loss and WD-exposure revealed similar functional repercussions. Endothelial ZEB2-loss eventually corrected WD-induced liver hypo-vascularization while ameliorating hepatic damage and steatosis. Thus, endothelial ZEB2-loss amplifies WD-induced LSEC fat metabolism and capillarization, while decreasing steatosis, in part through altered LSEC-LSEC communication. The disease-promoting role of endothelial ZEB2 in early MASLD as opposed to its protective role in fibrosis underscores a context-dependent effect in liver disease.

**Supplementary Information:**

The online version contains supplementary material available at 10.1038/s41598-025-05881-6.

## Introduction

Liver sinusoidal endothelial cells (LSECs) have special features adapted to the main liver functions, encompassing glucose and lipid storage/release, drug/toxin/pathogen clearance, and coagulation factor production^1–4^. To support their central function in bi-directional exchange between blood and liver parenchyma, they have fenestrae organized in sieve plates, lack an organized basement membrane, and express scavenger receptors^1,5,6^. This specialization is co-installed by LSEC-enriched transcription factors (TFs)^2,7–12^. During liver disease, LSECs lose sinusoidal characteristics and acquire a capillary EC phenotype, a process called capillarization^13^. Recently, we showed that TF Zinc-finger E-Box-binding Homeobox (ZEB)2 is important for LSEC specialization, since its loss results in capillarization and increased liver fibrosis^2,7^.

The most frequent form of chronic liver disease (CLD) is metabolic dysfunction-associated steatotic liver disease (MASLD; formerly known as non-alcoholic fatty liver disease), considered to be the hepatic manifestation of the metabolic syndrome^14–16^. MASLD represents a clinical spectrum, from steatosis to metabolic dysfunction–associated steatohepatitis (MASH), often combined with advanced fibrosis. Its initial stage, i.e., steatosis, encompasses excess hepatic triglyceride storage. LSEC-engagement during MASLD is poorly understood, but interesting – often contrasting – hypotheses exist^17^. First, Hammoutene and Rautou hypothesized that LSEC capillarization hampers trans-endothelial passive lipid transport, thereby releasing the brake on lipogenesis in hepatocytes and increasing liver fat content^3^. Indeed, lack of fenestrae, a capillarization hallmark, resulted in steatosis in mice deficient for plasmalemma vesicle-associated protein^18^. Mice with endothelial loss of Semaphorin 3a or Group IVa phospholipase A2 (encoded by *Pla2g4a*) showed attenuated loss of fenestrae in LSECs along with decreased steatosis^19,20^. Yet, loss of fenestrae has not always been observed in mouse models of MASLD^21^ and information in patients is scarce, with one study suggesting that loss of fenestrae would occur more prominently in early pre-MASH stages^22^. Second, single-cell RNA sequencing (scRNAseq) revealed that among organ ECs, capillary liver ECs were the second-most responsive to western-type diet (WD), after adipose tissue ECs, and the most responsive to diet reversal, suggesting a prominent role for LSECs during MASLD^23^. Third, while proliferative and angiogenic liver ECs have been described during MASLD^23–25^their impact on total liver vascularization and disease progression is unclear. On the other hand, dysfunctional ECs have been documented early during MASLD, before the onset of inflammation^26^. Finally, because peroxisome proliferator-activated receptor (PPAR)α elimination in hepatocytes aggravates steatosis^27^, PPAR(α) agonists were introduced as MASLD treatment^28^. PPAR signaling has been implicated in functional preservation in the systemic and hepatic vasculature^29,30^. However, it remains unknown how (much) these agonists affect LSEC behavior, since it was recently shown that PPAR(α) signaling is active in LSECs at baseline already and also upon lipid challenge^1,23,31,32^. Additionally, the benefits of modifying LSEC behavior for MASLD treatment need to be explored.

Given our observations that endothelial ZEB2 protects against LSEC capillarization and hepatic fibrosis^7^we wondered whether ZEB2 would also have a protective role during steatosis. Therefore, we exposed mice to WD and investigated the consequences of EC-specific *Zeb2* knockout for LSEC capillarization and steatosis.

## Results

### *Zeb2* is ubiquitously expressed in endothelium of healthy and fatty livers

**Fig. 1:**
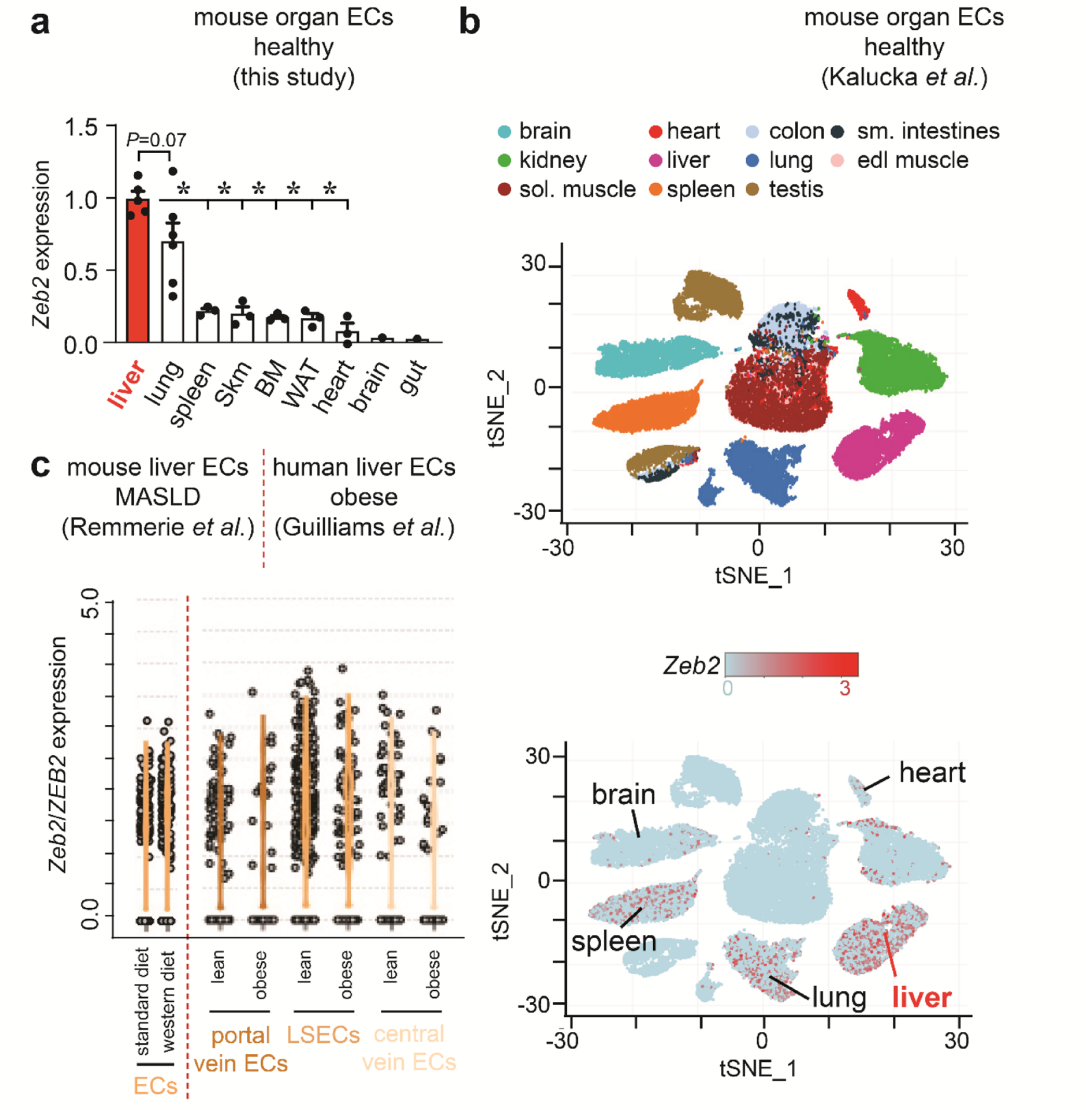
*Zeb2* is ubiquitously expressed in blood vascular endothelium of healthy and fatty livers. (**a**) *Zeb2* mRNA expression in endothelial cells (ECs) isolated by FACS from *EC*^*GFP*^ organs (*n* = 1–6). Skm: skeletal muscle; BM: bone marrow; WAT: white adipose tissue. (**b**) t-distributed Stochastic Neighbor Embedding (tSNE) plots extracted from Kalucka et al.^34^ showing the EC fraction of different organs of healthy adult mice (*top*) and their corresponding *Zeb2* mRNA expression (*bottom*). (**c**) Violin plots extracted from the scRNAseq dataset of the CD45-negative fraction from Remmerie et al.^36^ (left) showing *Zeb2* mRNA expression in liver ECs from mice exposed to standard (SD; *n* = 1) or western-type diet (WD, representing a pool of 2 mice, exposed to diet for 24 or 36 weeks) and extracted from Guilliams et al.^35^ (*right*) showing *ZEB2* mRNA expression in liver EC populations from lean and obese humans. LSECs: liver sinusoidal ECs; sol.: soleus; edl: extensor digitorum longus; sm.: small. Quantitative data are expressed as mean ± s.e.m; *P < 0.05 vs. indicated condition by one-way ANOVA with Tukey post-hoc test.

Our previous profiling analysis put forward ZEB2 as a TF ubiquitously expressed across the liver blood-vascular system and highly enriched in liver compared to heart and brain ECs^2,7^. For a more complete view on endothelial *Zeb2* expression, we expanded our analysis with extra-hepatic vascular beds from other metabolically demanding organs or organs with sinusoidal ECs and analyzed its expression in steatotic livers from patients with obesity or mice exposed to WD. Quantitative (q) real-time (RT)-PCR on ECs sorted from different organs of tamoxifen-inducible EC reporter mice newly generated for the current study by inter-crossing *Cdh5-Cre*^*ERT2*^ mice with mice carrying the RCE reporter (*EC*^*GFP*^)^33^ (Supplementary Fig.S1) and data-mining of the scRNAseq dataset from Kalucka et al.^34^ confirmed that liver endothelium is the highest *Zeb2* expresser among other sinusoidal ECs or ECs from metabolically demanding organs (Fig. 1a,b). Analysis of our WD-fed mice as well as scRNAseq datasets on mouse and human livers from Guilliams et al.^35^ and Remmerie et al.^36^ confirmed that *Zeb2* expression was ubiquitously detected in healthy liver endothelium, while WD-exposure/disease did not notably alter its expression (Fig. 1c; Supplementary Fig.S1). Altogether, endothelial *Zeb2* expression in the liver vasculature was ubiquitous and not influenced by (early) MASLD.

### *EC*^*Zeb2KO*^ amplifies WD-induced LSEC capillarization early during MASLD

We previously showed that endothelial ZEB2 preserves specialized features in LSECs after a fibrotic challenge^7^. To study the effect of endothelial ZEB2-loss on capillarization during MASLD, we used our EC-specific *Zeb2* knockout mice (*EC*^*Zeb2KO*^) mice in which *Zeb2* was specifically^7^ and to a comparable extent genetically inactivated in endothelium from healthy and steatotic mouse livers upon tamoxifen-treatment (Supplementary Fig.S1).

To induce MASLD, we exposed mice to WD, upon which *WT* mice (i.e., either tamoxifen-treated *Cre*-negative littermates (‘*EC*^*WT*^ ‘) or *EC*^*GFP*^ reporter mice; Supplementary Fig.S1; Supplementary NoteS1) progressively gained weight and developed steatosis from 4 weeks (w) onwards (Supplementary Fig.S1 + S2). After 24w of WD, steatosis was highly prominent while inflammation and parenchymal fibrosis were limited, suggesting that mice were still in the early MASLD stage, mainly featuring steatosis (Supplementary Fig.S2). Indeed, initial WD-induced increases in *Ptprc* and *Adgre1* total liver mRNA levels were small and not reflected by the number of CD45^+^ inflammatory cells in situ (Supplementary Fig.S2). Inflammation and fibrosis parameters were not significantly altered by endothelial ZEB2 loss (Supplementary Fig.S2). Thus, our model was most suitable to study the role of endothelial ZEB2 in steatosis.

**Fig. 2:**
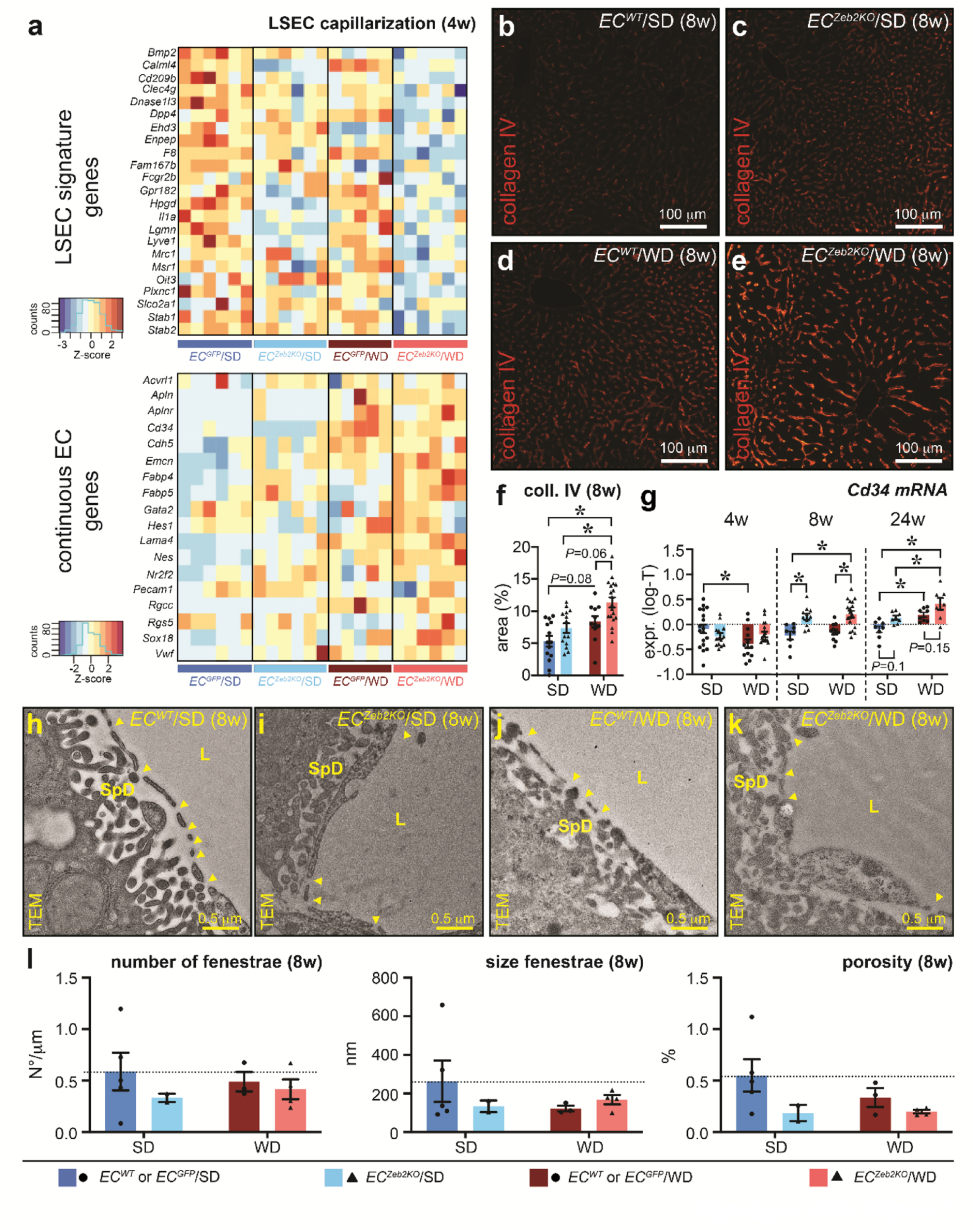
*EC*^*Zeb2KO*^ amplifies WD-induced LSEC capillarization early during MASLD. (**a**) Heatmaps with genes in alphabetical order showing color-coded z-scores of normalized RNAseq data of liver sinusoidal EC (LSEC) signature genes (*top*) and continuous EC genes (*bottom*) in LSECs sorted from mouse livers belonging to the indicated condition after 4 weeks (w) of standard (SD) or western-type diet (WD). (**b**–**f**) Liver cross-sections of *EC*^*WT*^ (**b**,**d**) or *EC*^*Zeb2KO*^ (**c**,**e**) mice after 8w of SD (**b**,**c**) or WD (**d**,**e**) stained for collagen (coll.)-type IV (red) and corresponding quantification (**f**) of collagen type IV expression presented as area % (*n* = 10 *EC*^*WT*^/4 *EC*^*GFP*^/14 *EC*^*Zeb2KO*^ for SD; *n* = 11 *EC*^*WT*^/0 *EC*^*GFP*^/19 *EC*^*Zeb2KO*^ for WD). (**g**) mRNA expression (presented as log-transformed or ‘log-T’ data) of capillarization marker *Cd34* in whole livers from *EC*^*WT*^/*EC*^*GFP*^ or *EC*^*Zeb2KO*^ mice after 4w (*n* = 4 *EC*^*WT*^/14 *EC*^*GFP*^/14 *EC*^*Zeb2KO*^ for SD; *n* = 0 *EC*^*WT*^/13 *EC*^*GFP*^/18 *EC*^*Zeb2KO*^ for WD), 8w (*n* = 5 *EC*^*WT*^/3 *EC*^*GFP*^/11 *EC*^*Zeb2KO*^ for SD; *n* = 11 *EC*^*WT*^/0 *EC*^*GFP*^/18 *EC*^*Zeb2KO*^ for WD) and 24w (*n* = 3 *EC*^*WT*^/5 *EC*^*GFP*^/8 *EC*^*Zeb2KO*^ for SD; *n* = 4 *EC*^*WT*^/4 *EC*^*GFP*^/7 *EC*^*Zeb2KO*^ for WD) of SD or WD. (**h**–**l**) Representative transmission electron microscopy (TEM) images showing fenestrae (indicated by yellow arrowheads) in sinusoids of *EC*^*WT*^ (**h**,**j**) or *EC*^*Zeb2KO*^ (**i**,**k**) mice after 8w of SD (**h**,**i**) or WD (**j**,**k**) and corresponding quantification of the average number of fenestrae (**l**, *left*), size of fenestrae (**l**, *middle*) and porosity (**l**, *right*; *n* = 1 *EC*^*WT*^/4 *EC*^*GFP*^/2 *EC*^*Zeb2KO*^ for SD; *n* = 1 *EC*^*WT*^/2 *EC*^*GFP*^/4 *EC*^*Zeb2KO*^ for WD). SpD: Space of Disse; L: lumen. Quantitative data are expressed as mean ± s.e.m. *: *P* < 0.05 vs. indicated condition by two-way ANOVA with Tukey post-hoc test. Pictures in (**b**–**e**) were taken with an EC Plan-Neofluar 10x/0.30 M27 objective on a Zeiss Axio Imager Z1 equipped with an AxiocamMRc5 and Axiovision software. Pictures in (**h**–**k**) were taken with a JEOL JEM1400 microscope.

Capillarization signs were already manifest in this early stage, as evident from RNAseq on eGFP^+^ LSECs from *EC*^*Zeb2KO*^ and *EC*^*GFP*^ livers fed a standard diet (SD) or WD for 4w (Fig. 2a). Relative expression levels of landmark genes of continuous ECs and other liver cell-types demonstrated the purity and sinusoidal identity of the sorted LSEC populations (Supplementary Fig.S3). One sample from the *EC*^*GFP*^/WD condition was omitted in subsequent analyses based on insufficient quality (Supplementary Fig.S3). All 12 mice from the *EC*^*Zeb2KO*^/WD and *EC*^*Zeb2KO*^/SD conditions showed successful *exon 7* deletion (Supplementary Fig.S3).

Plotting the expression of a selected gene-set in a heatmap revealed an overall decreased LSEC signature gene expression upon endothelial ZEB2-loss, and WD-exposure alone had a partially similar effect. Simultaneously, *EC*^*Zeb2KO*^ increased continuous EC marker expression in LSECs, including *Vwf*. Endothelial ZEB2-loss and WD-exposure together boosted these effects (Fig. 2a). Accordingly, at 8w of diet, VWF antigen plasma levels were significantly increased by the combined challenge (105% vs. 58% of reference plasma pool levels; *P* < 0.05) compared to SD/*EC*^*WT*^. Immunofluorescence staining for collagen-type IV revealed increased basement membrane formation triggered by WD, which was amplified upon combined challenge of *EC*^*Zeb2KO*^ and WD-feeding (Fig. 2b-f). QRT-PCR on whole-liver RNA revealed an increase in capillarization by the combined challenge, with gradual increase of capillarization marker *Cd34* mRNA^37^ from 8w of diet onwards (Fig. 2g). Transmission electron microscopy (TEM) at 8w revealed that fenestrae were still observed in all challenged conditions (Fig. 2h-k). Quantitative assessment of fenestrae revealed a downward trend in their number and size, resulting in a lower porosity upon endothelial ZEB2 loss and/or WD-exposure (Fig. 2l). Altogether, endothelial ZEB2-loss and WD-exposure cooperated in causing increased sinusoidal capillarization.

### *EC*^*Zeb2KO*^ and WD-exposure both induce LSEC proliferation early during MASLD

**Fig. 3:**
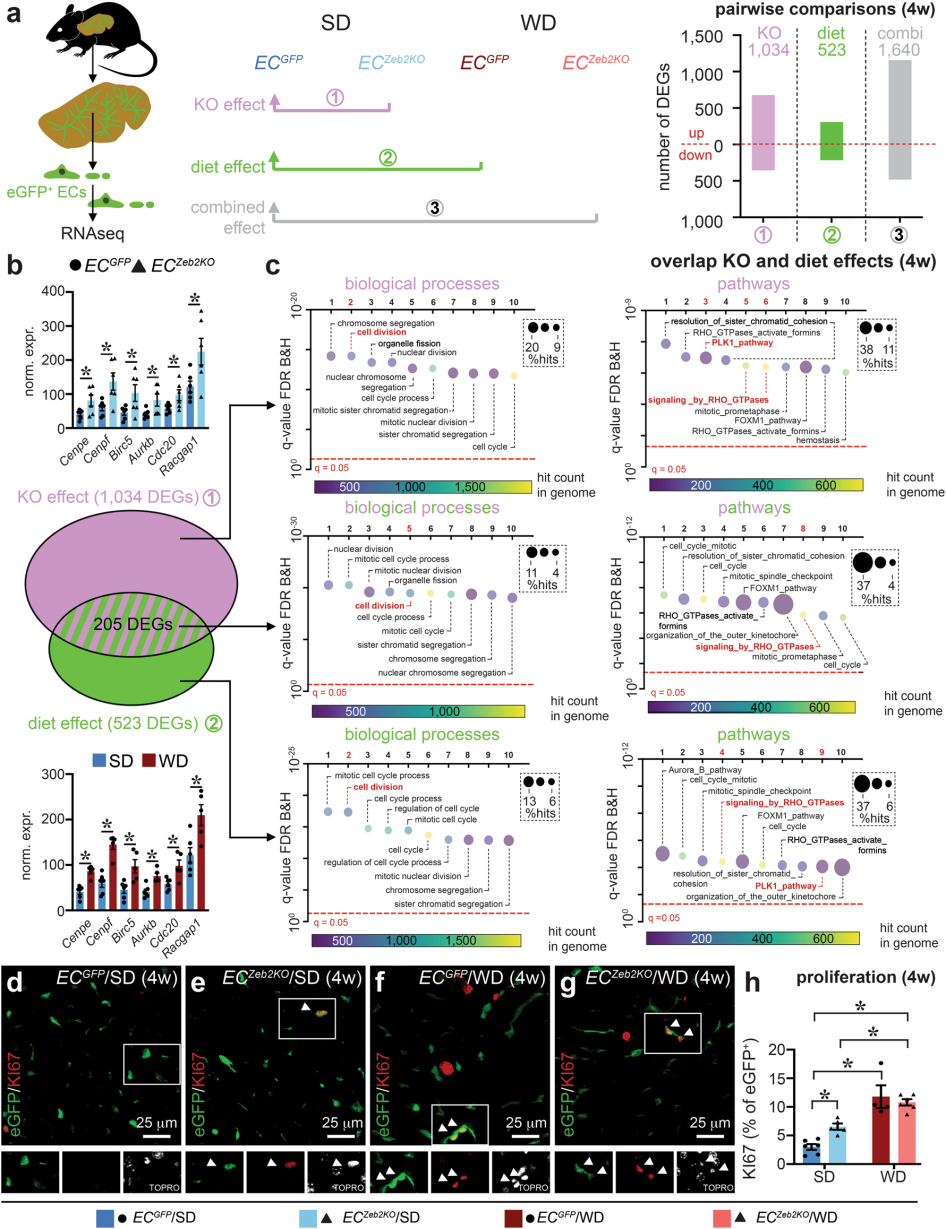
*EC*^*Zeb2KO*^ and WD-exposure both induce LSEC proliferation early during MASLD. (**a**) Schematic diagram of the RNAseq set-up (*left*), overview of (3) pairwise comparisons (*middle*) and quantification of the number of differentially expressed genes (DEGs) per comparison (*right*) at 4 weeks (w) of standard diet (SD) or western-type diet (WD). A red dotted line separates the up-and downregulated number of DEGs (total number is indicated on top of bars). Combi: combined challenge. (**b**) Venn diagram (*middle*) showing overlap (205 DEGs with expression changes in the same direction) between DEGs from the knockout (KO; comparison 1 in purple) and the diet effect (comparison 2 in green) and expression of representative DEGs (normalized data; corresponding to functional terms highlighted in red in panel (**c**); *n* = 5–6) extracted from comparison 1 (*top*) and 2 (*bottom*). (**c**) Functional annotation analysis on the combined up- and downregulated DEGs. Bubble plots represent the top 10 functional terms (biological processes on the *left*, pathways on the *right*) ranked according to false discovery rate (FDR) related to comparison 1 (*top*), comparison 2 (*bottom*) and the overlap between comparison 1 and 2 (*middle*). Significance level in (**c**) indicated by red dotted lines. All quantitative data are also shown in Supplementary Table 1. (**d**-**h**) Liver cross-sections of *EC*^*GFP*^ (**d**,**f**) or *EC*^*Zeb2KO*^ (**e**,**g**) mice after 4w of SD (**d**,**e**) or WD (**f**,**g**) co-stained for eGFP (green) and KI67 (red) and corresponding quantification (**h**) of proliferating cells (indicated by white arrowheads) expressed as % KI67^+^ cells (*n* = 4–6). Single-color images of the delineated inset are shown below each panel. Quantitative data are expressed as mean ± s.e.m; Panel (**b**): *: FDR (false discovery rate) < 0.05 vs. indicated condition by differential gene expression analysis with applying negative binomial general linear model approach and Benjamini-Hochberg multiple testing correction. Panel (**h**) *: *P* < 0.05 by two-way ANOVA with Tukey post-hoc test. Pictures in (**d–g**) were taken with a Plan-Apochromat 40x/1.3 Oil DIC M27 objective on a Zeiss LSM 700 AxioObserver Z1 equipped with an LSM T-PMT detector and ZEN software.

To gain more unbiased insight into changes caused by *EC*^*Zeb2KO*^ and/or WD-feeding, we performed three pairwise analyses revealing differentially expressed genes (DEGs; Fig. 3a; Supplementary Fig.S4). First, *EC*^*Zeb2KO*^ had a more dramatic and uniform effect on LSEC expression profiles than WD-exposure. Indeed, the *EC*^*Zeb2KO*^ *vs. EC*^*GFP*^ condition (comparison 1) had twice the number of DEGs and a clearer segregation on clustering heatmaps and principal component analysis plots compared to the WD vs. SD condition (comparison 2; Fig. 3a; Supplementary Figs.S3 + S4; Table 1). As expected from its predominant role as transcriptional repressor^38^a majority of DEGs (65.5%) was upregulated by *EC*^*Zeb2KO*^ (Fig. 3a; Supplementary Fig.S4; Table 1). Similarly, WD-exposure mostly caused gene expression upregulation (58.7% of DEGs; Fig. 3a; Supplementary Fig.S4; Table 1). Remarkably, from the 523 DEGs elicited by WD-feeding in *EC*^*GFP*^ mice, a considerable number (41%) were also affected by *EC*^*Zeb2KO*^ upon SD-feeding (Fig. 3b; Supplementary Table 1).

Second, functional annotation on the combined up- and downregulated DEGs within comparison 1 revealed that the dominating biological processes/pathways altered by *EC*^*Zeb2KO*^ were related to proliferation (‘cell division’, ‘RHO-GTPase’ or ‘PLK1_pathway’; Fig. 3c; Supplementary Table 1). As expected by the large overlap in DEGs with the effect of *EC*^*Zeb2KO*^the dominating biological processes/pathways represented by DEGs caused by WD-exposure (comparison 2) were similarly related to proliferation (Fig. 3c; Supplementary Table 1). Accordingly, biological processes/pathways emerging from functional annotation analysis on genes commonly altered by *EC*^*Zeb2KO*^ and WD-exposure were equally related to proliferation (Fig. 3c; Supplementary Table 1). Separate functional annotation on the up- or downregulated DEGs revealed that the common proliferation-related terms emerged from the upregulated DEG sets, while the downregulated DEGs revealed some differences between genotype and diet effects, the former affecting ‘leukocyte migration’ and the latter ‘angiogenesis regulation’ (Supplementary Fig.S5 + Table 1). Thus, *Zeb2* inactivation in ECs and WD-exposure both triggered proliferative gene expression in LSECs. The net effect of proliferation-related gene expression changes was an increase in proliferation confirmed in situ by the increased KI67^+^ cell-fraction within the eGFP^+^ LSEC population compared to the unchallenged condition. Altogether, *EC*^*Zeb2KO*^ and WD-feeding both provoked a proliferative response in LSECs, however, there was no additive effect of the combined challenge (Fig. 3d-h).

### *EC*^*Zeb2KO*^ and WD-exposure interact in affecting endothelial lipid metabolism

**Fig. 4:**
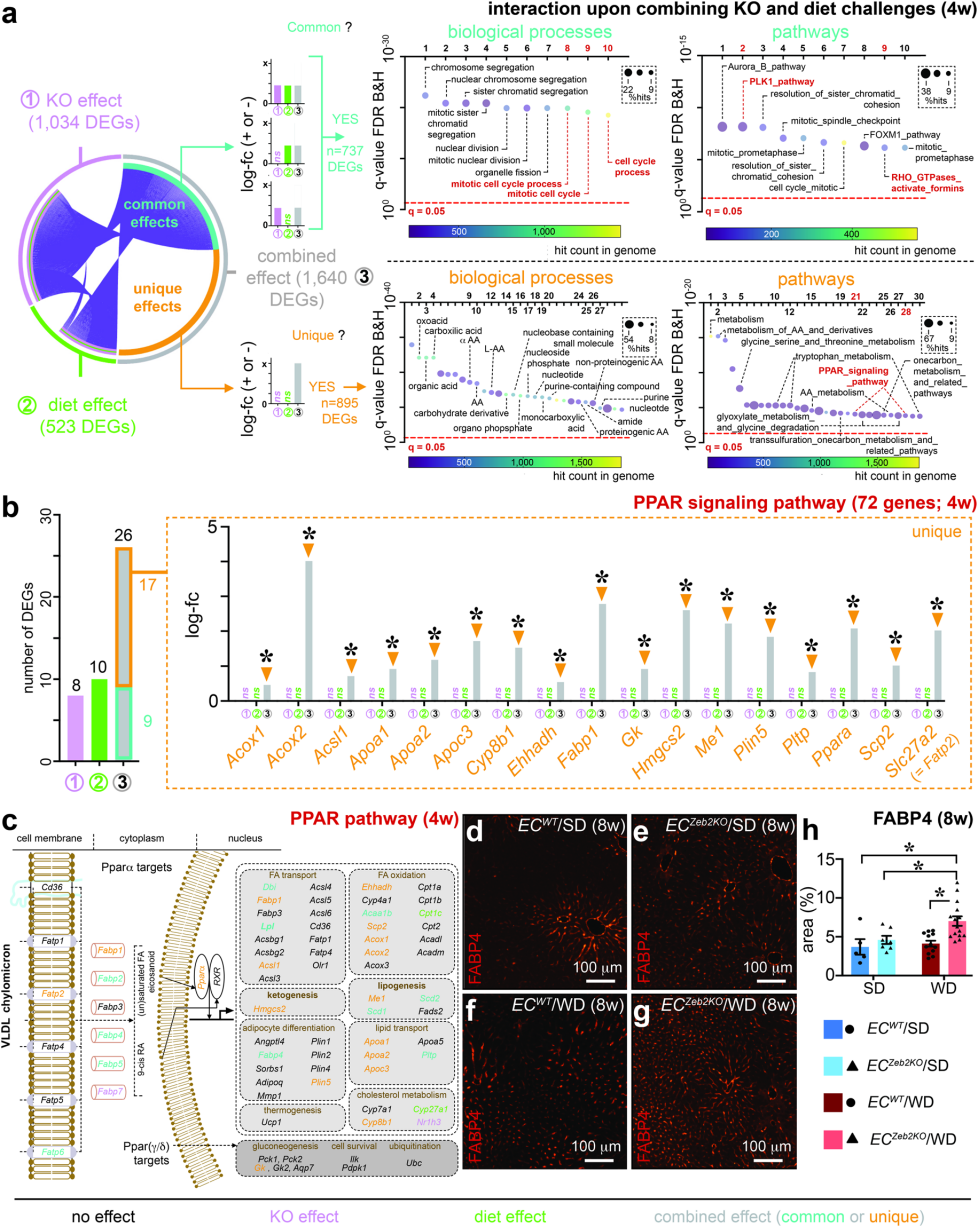
*EC*^*Zeb2KO*^ and WD-exposure interact to affect endothelial lipid metabolism. (**a**) Circos plot (*left*) showing overlap (‘common’, i.e., genes with expression changes in the same direction; turquoise) or not (‘unique’; orange) between DEGs from comparison 1 or 2 (single challenges: purple or green) and comparison 3 (combined challenge: gray). Schematic expression patterns for common or unique genes across single and combined challenges are shown next to the circos plot. Functional terms derived from the combined up- and downregulated DEGs (full list: see Supplementary Table 1 for common (*top*) or unique (*bottom*) DEGs are plotted as bubble plots representing top 10 or 30 biological processes (*middle*) and pathways (*right*) ranked according to false discovery rate (FDR). Pathways of interest are highlighted in red. Significance level in bubble plots is indicated by red dotted lines. (**b**) Quantitative analysis of DEGs from the functional term ‘PPAR_signaling_pathway’ showing number of genes that emerged as differentially expressed from comparison 1, 2 or 3 (*left*) and expression changes (expressed as log-fc) for DEGs with a ‘unique’ profile (indicated by arrowheads; *right*). *P < 0.05 vs. no challenge. Statistical analyses were performed according to pairwise comparisons related to single or combined challenges as indicated. Ns: not significant. (**c**) Schematic representation (*bottom-left*) shows cellular localization and function of all 72 PPAR signaling pathway genes color-coded according to their DEG status (black: not differentially expressed; purple or green: DEG from comparison 1 or 2, respectively; orange or turquoise: unique or common DEG from comparison 3). (**d**–**h**) Liver cross-sections of *EC*^*WT*^ (**d**,**f**) or *EC*^*Zeb2KO*^ (**e**,**g**) mice after 8 weeks (w) of SD (**d**,**e**) or WD (**f**,**g**) stained for FABP4 (red) and corresponding quantification (**h**) of FABP4 expression plotted as area % (*n* = 5–15). Quantitative data are expressed as mean ± s.e.m. *: *P* < 0.05 vs. indicated condition by two-way ANOVA with Tukey post-hoc test. Pictures in (**d**–**g**) were taken with an EC Plan-Neofluar 10x/0.30 M27 objective on a Zeiss Axio Imager Z1 equipped with an AxiocamMRc5 and Axiovision software. Data in all panels, except for (**d**–**h**) correspond to 4w of diet exposure. VLDL: very low-density lipoprotein, RA: retinoic acid, FA: fatty acid.

After we showed that the combination of *EC*^*Zeb2KO*^ and WD-feeding boosted gene expression changes reflecting LSEC capillarization, we investigated the interaction between both challenges in a more unbiased way by performing a whole-genome pairwise comparison (comparison 3), i.e. the combined challenge vs. no challenge. The resulting DEG list contained 1,640 genes, 70.5% of which were upregulated upon combined challenge (Fig. 3a; Supplementary Fig.S4; Table 1). Notably, overlap between the compiled DEG list from the single challenges with that of the combined challenge was only ∼45% (737 common DEGs; Fig. 4a; Supplementary Table 1). In accordance with the common effect of each challenge on cell proliferation (Fig. 3b-h; Supplementary Fig.S5), the dominating annotated biological processes and pathways of the 737 common up- and downregulated DEGs were related to ’cell cycle’ and ‘PLK1_pathway/RHO GTPase’, respectively (Fig. 4a; Supplementary Table 1). On the other hand, *EC*^*Zeb2KO*^ and WD-feeding interacted to induce a substantial amount of unique expression changes (895 unique DEGs; Fig. 4a; Supplementary Table 1). Functional annotation on the combined up- and downregulated unique DEGs revealed biological processes and pathways involving ‘(lipid) metabolism’ and ‘PPAR_signaling_pathway’ (Fig. 4a; Supplementary Table 1). Separate functional annotation on the up- or downregulated DEGs revealed that the common proliferation-related terms and the unique metabolism/PPAR-related terms emerged from the upregulated DEG sets, while the downregulated DEGs were too few to retrieve a sufficient number of significant terms (Supplementary Fig.S6 + Table 1). The combined challenge substantially extended the common effect on ‘PPAR signaling’. From the 26 DEGs related to ‘PPAR signaling’, 17 were uniquely induced by the combined challenge (Fig. 4b; Supplementary Table 1). The 26 common and unique genes together widely represented biological processes known to be regulated by PPAR signaling, including lipogenesis, lipid/fatty acid (FA) transport, FA oxidation, ketogenesis and cholesterol metabolism (Fig. 4c)^39^. Accordingly, FA binding protein (FABP)4 protein expression was upregulated in LSECs of WD-exposed livers, most notably those simultaneously lacking endothelial ZEB2*-*expression (Fig. 4d-h). Altogether, *EC*^*Zeb2KO*^ and WD-challenge significantly interacted in boosting LSEC lipid metabolism.

### *EC*^*Zeb2KO*^ and WD-exposure interact in altering LSEC-LSEC communication during MASLD

**Fig. 5:**
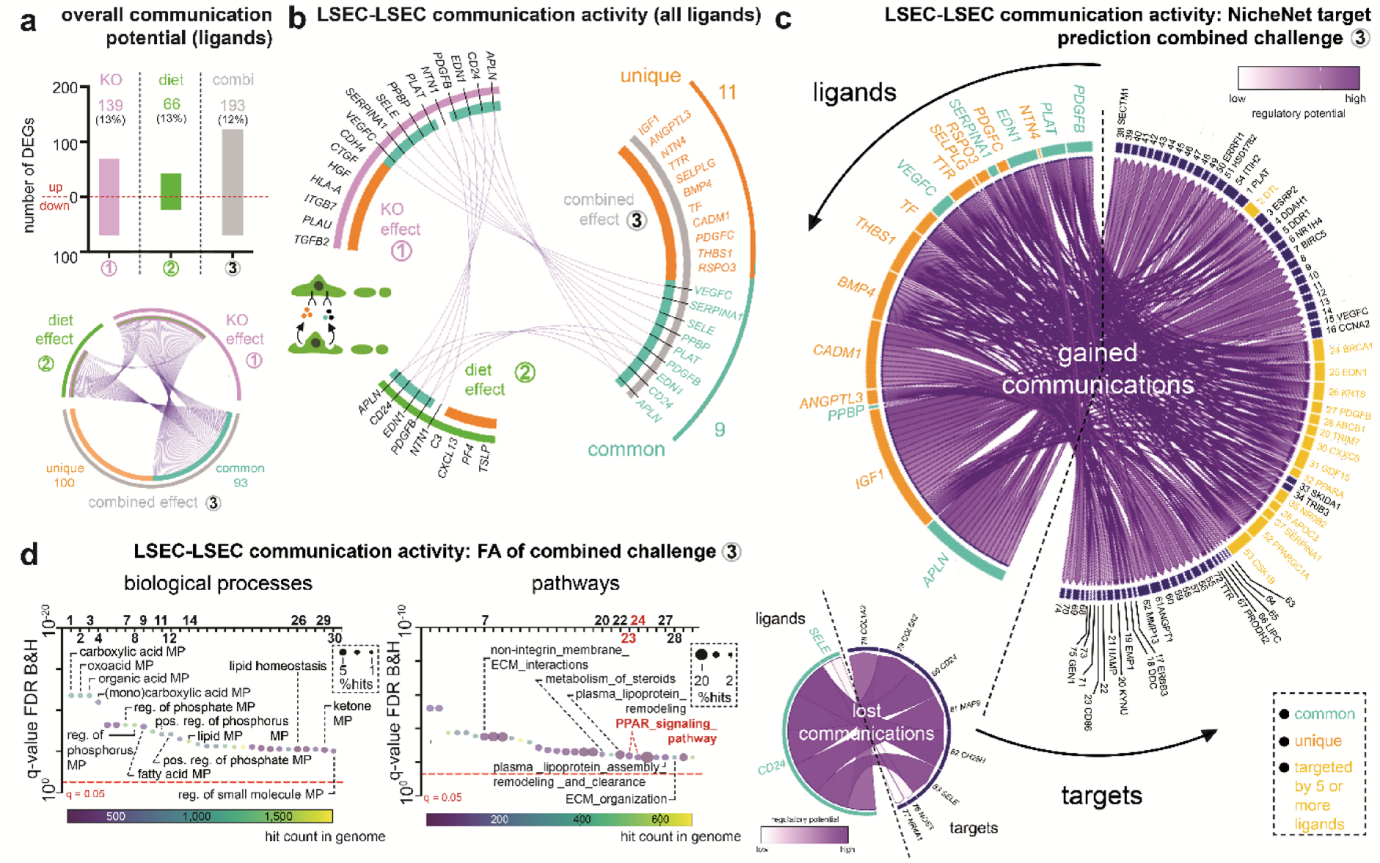
*EC*^*Zeb2KO*^ and WD-exposure interact in altering LSEC-LSEC communication during MASLD. (**a**) Diagram showing quantification of the number of differentially expressed genes (DEGs) encoding highly likely secreted proteins per comparison (*top*). A red line separates the up-and downregulated number of DEGs (total number is indicated on top of bars). Combi: combined challenge; SD: standard diet; WD: western-type diet. The *bottom* panel shows a circos plot representing overlap (turquoise; 93 DEGs representing common genes) or not (‘unique’; orange; 100 DEGs) between DEGs from comparison 1 or 2 (single challenges in purple and green, respectively) and comparison 3 (combined challenge in gray). (**b**) Circos plot based on the RNAseq dataset generated in the current paper summarizing communication activity based on LSEC ligands communicating with LSECs extracted by NicheNet and representing overlap (turquoise; 9 ligands representing common genes in the combined challenge compared to the single challenges) or not (orange; 11 unique ligands) between upregulated ligands from comparison 1 or 2 (single challenges in purple and green, respectively) and comparison 3 (combined challenge in gray). (**c**) NicheNet plots showing predicted targets downstream of (unique in orange or common in turquoise) ligands that were altered upon the combined knockout and diet challenge. The large plot corresponds to ligands (*n* = 18) upregulated by the combined challenge (i.e., gained communications), the small plot corresponds to ligands (*n* = 2) downregulated by the combined challenge (i.e., lost communications). Targets that are connected to at least 5 ligands are highlighted in yellow. Target numbers correspond to those shown in Supplementary Fig.S8. (**d**) Bubble plots representing the top 30 functional terms (biological processes on the *left*, pathways on the *right*) ranked according to false discovery rate (FDR) related to all (common and unique) ligands + associated target genes from panel (**c**). A full list of top 30 functional terms is shown in Supplementary Table 2. Significance levels are indicated by red dotted lines. MP: metabolic process.

LSEC communication with other liver cell-types is an important determinant of MASLD development^40^. Therefore, by overlap with a compiled list of 2,356 genes encoding highly likely secreted proteins^41^we first identified within our DEG lists ~ 13% genes encoding proteins that potentially interact with neighboring cells (Fig. 5a; Supplementary Table 2). WD-exposure and ZEB2-loss in ECs triggered changes in the expression of these genes, with significant interaction between both challenges, similar to the overall gene expression changes (Figs. 3a and 5a). Indeed, 93 genes were commonly regulated by at least one of the challenges and the combined challenge, while about 100 genes were uniquely regulated by the combined challenge (Supplementary Table 2). Thus, like for the whole gene-set, *EC*^*Zeb2KO*^ and WD-challenge significantly interacted in determining expression alterations of genes encoding secreted products.

To identify the preferential target cell-type(s) of LSEC-secreted products during MASLD, we used Multi-NicheNet to predict communication activity using available scRNAseq data of mice exposed to SD or WD (Supplementary Fig.S7)^35^. We considered both WD-induced gain and loss in communication by looking at up- or downregulated ligands in LSECs, respectively, for which the predicted receptors are expressed in the receiver cell. Collectively, WD-exposure had a substantial effect on LSEC-LSEC and LSEC-Kupffer cell communication compared to LSEC communication with hepatocytes or hepatic stellate cells. This was evident from a high number of lost or gained ligand-receptor interactions and resulting altered target genes (Supplementary Fig.S7).

We next zoomed in on LSEC-LSEC communication and used NicheNet on our own dataset to filter out those ligands that likely give rise to functional communication activity based on corresponding receptor and altered target gene expression in receiving LSECs (Supplementary Fig.S8). Upon *Zeb2* genetic inactivation, WD-exposure, or the combination of both, several ligands were predicted to give rise to altered target expression in LSECs (Fig. 5b). As above, we evaluated gain or loss in communication by screening for ligands up- or downregulated, respectively, by the challenge. Again, there was substantial overlap in predicted active ligands between single and combined challenges (Fig. 5b ‘common’; Supplementary Fig.S8). However, 11 out of 20 ligand alterations in communication were uniquely invoked by the combined challenge (Fig. 5b). Eighteen out of 20 ligands were upregulated by the combined challenge and gave rise to a gain in communication (Fig. 5c). Unique and common ligands together were linked to 83 significantly altered target genes (Fig. 5c; Supplementary Fig.S8). Fifteen targets, including PPARα signaling pathway members *Ppara* and *Apoc3* and vasoconstrictor *endothelin-1* (*Edn1*) were triggered by minimally 5 ligands.

Functional annotation of all 20 ligands and 83 associated predicted targets revealed biological processes/pathways, like terms related to extracellular matrix (ECM) and metabolism/PPAR (Fig. 5d; Supplementary Table 2). When splitting up the annotation between gained and lost communications, the metabolism/PPAR terms were represented in the gained communications, while on the lost communications side other terms emerged, including ‘ECM/collagen synthesis/degradation’, ‘leukocyte migration’ and ‘erythrocyte aggregation’ (Supplementary Fig.S9 + Table 2). Altogether, this suggests that some processes, such as metabolism and PPAR(α) signaling, identified earlier within our RNAseq dataset, were caused at least partially by altered LSEC-LSEC communication.

### *EC*^*Zeb2KO*^ rescues WD-induced liver damage, steatosis and hypo-vascularization

**Fig. 6:**
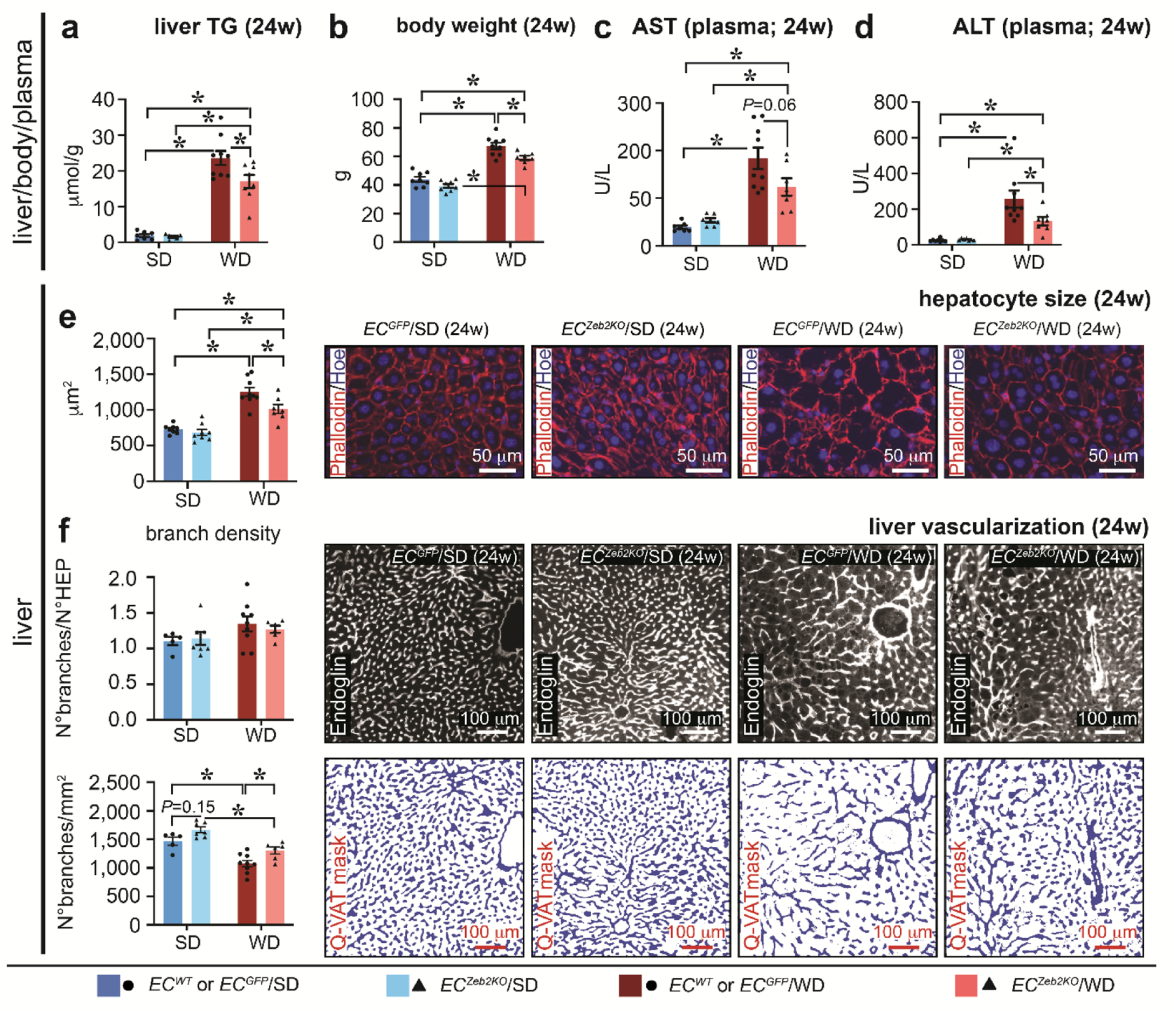
*EC*^*Zeb2KO*^ attenuates WD-induced liver damage, steatosis and hypo-vascularization. (**a–d**) Quantification of liver triglyceride levels (**a**; *n* = 2 *EC*^*WT*^/5 *EC*^*GFP*^/6 *EC*^*Zeb2KO*^ for SD; *n* = 4 *EC*^*WT*^/5 *EC*^*GFP*^/7 *EC*^*Zeb2KO*^ for WD), body weight (**b**; *n* = 3 *EC*^*WT*^/5 *EC*^*GFP*^/8 *EC*^*Zeb2KO*^ for SD; *n* = 4 *EC*^*WT*^/5 *EC*^*GFP*^/7 *EC*^*Zeb2KO*^ for WD), plasma liver enzyme levels (**c**: aspartate transaminase (AST, *left*; *n* = 2 *EC*^*WT*^/5 *EC*^*GFP*^/7 *EC*^*Zeb2KO*^ for SD; *n* = 4 *EC*^*WT*^/5 *EC*^*GFP*^/7 *EC*^*Zeb2KO*^ for WD) and; **d**: alanine transaminase (ALT, *right*; *n* = 3 *EC*^*WT*^/5 *EC*^*GFP*^/7 *EC*^*Zeb2KO*^ for SD; *n* = 4 *EC*^*WT*^/5 *EC*^*GFP*^/7 *EC*^*Zeb2KO*^ for WD) of mice from the indicated conditions after 24 weeks (w) of standard diet (SD) or western-type diet (WD). (**e**) Diagram showing quantification of hepatocyte size after 24w of SD or WD (*left*). The *right* inset panels show images of liver cross-sections stained with Rhodamine-Phalloidin to label cell contours for all 4 conditions (*n* = 3 *EC*^*WT*^/5 *EC*^*GFP*^/8 *EC*^*Zeb2KO*^ for SD; *n* = 4 *EC*^*WT*^/5 *EC*^*GFP*^/7 *EC*^*Zeb2KO*^ for WD). Nuclei are counterstained with Hoechst (blue). (**f**) Quantification of liver vascular branch density (expressed as branch/hepatocyte ratio (*top left*) or number/area in mm^2^ (*bottom left*)) after 24w of SD or WD (*n* = 3 *EC*^*WT*^/2 *EC*^*GFP*^/7 *EC*^*Zeb2KO*^ for SD; *n* = 4 *EC*^*WT*^/5 *EC*^*GFP*^/6 *EC*^*Zeb2KO*^ for WD). The inset panels on the *right* show representative images of ENDOGLIN-stained cross-sections (white; *top*) and corresponding vascular masks (blue; *bottom*) generated by Q-VAT (Quantitative Vascular Analysis Tool) software for all 4 conditions. Quantitative data are expressed as mean ± s.e.m; *P < 0.05 vs. indicated condition by two-way ANOVA with Tukey post-hoc test. Statistical testing for ALT was performed on the log-transformed data. Pictures in *e* were taken with an EC Plan-Neofluar 20x/0.50 M27 objective on a Zeiss Axio Imager Z1 equipped with an AxiocamMRc5 and Axiovision software. Pictures in *f* were taken with a Plan Apo 20x (NA 0.75) objective on a Nikon Eclipse Ni-E with Marzhauser Slide Express 2 equipped with a Hamatsu Orca Flash 4.0 camera and NIS Elements software.

We subsequently wondered whether changes in endothelial expression and LSEC-LSEC communication triggered by *EC*^*Zeb2KO*^ and WD-exposure had repercussions for steatosis progression and liver vascularization. In accordance with previous observations^42^, WD-exposure significantly increased cholesterol and downregulated triglycerides in plasma (Supplementary Fig.S10). While WD-feeding invoked hepatic triglyceride accumulation in both genotypes, 24w of WD-feeding caused a significantly higher increase in *WT* livers (Fig. 6a). In addition, WD-induced body weight was significantly lower in mice lacking endothelial ZEB2 by 24w (Fig. 6b). Furthermore, liver parenchyma integrity was compromized after 24w of WD-exposure, as evident by increased plasma liver enzymes (Fig. 6c,d). In accordance with the hepatic triglyceride content, liver damage and hepatocyte hypertrophy were also attenuated in livers lacking endothelial ZEB2 (Fig. 6c–e; Supplementary Fig.S10).

While it has been hypothesized that capillarization triggers a compensatory increase in hepatic lipogenesis^3^the increased capillarization upon endothelial ZEB2-loss did not provoke more hepatic lipid production. Indeed, 4w of WD similarly induced whole liver expression of lipogenesis regulator *Srebf1*. At 24w, WD-induced levels of genes encoding lipogenesis enzymes *Fasn* and *Acaca* were not significantly affected by ZEB2-loss (Supplementary Fig.S10).

To evaluate in a representative and global manner whether and how ZEB-2 loss and/or WD-exposure affect liver vascularization, we used Q-VAT (Supplementary Fig.S11)^43^. While WD-exposure did not significantly alter the capillary/hepatocyte ratio, vascular branch density per parenchymal area was significantly decreased at 24w of WD in accordance with the increased hepatocyte size in *WT* livers (Fig. 6e,f). This hypo-vascularization suggests that increased proliferation upon 4w of WD-exposure was not sufficient to compensate for the expanding liver (Figs. 3d-h and 6e,f). In contrast, in *EC*^*Zeb2KO*^ livers, vascular branch density per parenchymal area was partially restored, again in accordance with the attenuated WD-induced hepatocyte hypertrophy (Fig. 6e,f). Altogether, endothelial ZEB2-loss ameliorated WD-induced liver steatosis, damage and hypo-vascularization.

## Discussion

MASLD is becoming the most prevalent CLD due to the ongoing obesity epidemic. Since fibrosis is the etiology-independent result of CLD, most studies, including those related to MASLD, have focused on understanding the mechanisms that orchestrate excessive collagen deposition^44^. Nevertheless, before the challenge evokes a fibrotic response, there are other connected pathogenic mechanisms. Understanding initial events is important not only for early therapeutic intervention, but also to diagnose MASLD before reaching advanced stages. In MASLD, excessive lipid accumulation initiates hepatocyte damage.

**Fig. 7:**
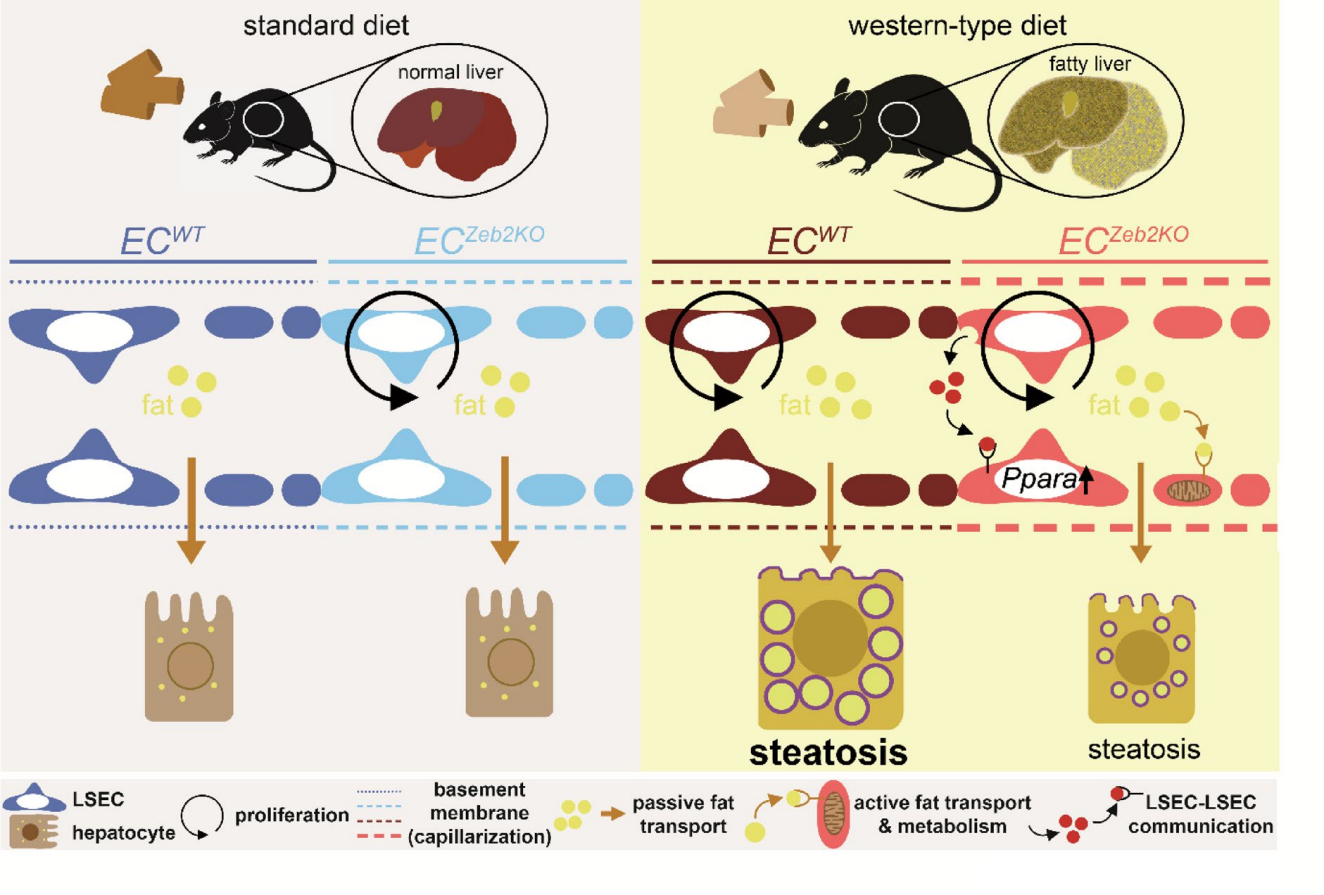
Graphical summary. Mice deficient for ZEB2 specifically in their endothelial cells (*EC*^*Zeb2KO*^), and their *wild-type* control littermates (*EC*^*WT*^), were randomized to standard or western-type diet (WD), the former to look at normal conditions, the latter to mimic the fatty liver stage of metabolic dysfunction-associated steatotic liver disease. Pairwise comparisons of these four conditions revealed that exposure to WD caused genotype-dependent or independent changes in liver sinusoidal endothelial cells (LSECs), more specifically in capillarization, proliferation, and also expression profiles including *Ppara*, the latter uniquely induced by combined *EC*^*Zeb2KO*^ and WD challenge. The most prominent change in the absence of endothelial ZEB2 was the induction of gene expression related to active lipid transport and metabolism in LSECs governed by LSEC-to-LSEC PPARα-dependent signaling, hypothetically resulting in reduced fat transfer to hepatocytes and less steatosis.

Here, we identified endothelial ZEB2 as a multi-factorial instigator of steatosis, thereby down-tuning PPAR(α) signaling, partly through altered communication among LSECs (Fig. 7). Because endothelial ZEB2-loss also hampered the WD-induced body weight increase, we cannot exclude an effect of endothelial ZEB2 deficiency on whole body metabolism and/or calorie intake. Furthermore, since ECs from certain extrahepatic lipid-handling organs (such as WAT) also express low ZEB2 levels, we cannot rule out indirect effects on hepatic lipid handling. Although WD-induced adipocyte hypertrophy occurred to a similar extent in both genotypes (Supplementary Fig.S12), excluding an indirect role for ZEB2 in adipose tissue ECs would require a more in-depth assessment of adipose tissue (metabolic) function. The second-highest expression level for endothelial ZEB2 was found in the lung where its loss may potentially affect lung function (Fig. 1a). A recent study found an inverse correlation between lung function and the risk of developing MASLD^45^. Nevertheless, to exclude the involvement of extrahepatic endothelial ZEB2, an approach where ZEB2 is specifically deleted in liver ECs is required. Despite endothelial ZEB2’s multi-factorial effect on steatosis, short- or long-term WD-exposure did not notably alter *Zeb2* expression in liver ECs. In contrast, GATA4, another TF enriched in LSECs, did not affect hepatocyte triglyceride content in a MASLD model^46^. Although endothelial ZEB2 counteracted capillarization during both steatosis and fibrosis^7^it was only protective against fibrosis, partly through altered communication with hepatic stellate cells. Thus, TFs involved in LSEC differentiation have different effects on the development of steatosis, and ZEB2 has a challenge-type dependent role in liver disease.

Capillarization has been causally linked to fibrosis in several liver disease models, in part through allowing hepatic stellate cell activation^44^. A causal role for LSEC capillarization in MASLD has also been suggested in studies showing that it occurs before the onset of steatosis^42^ as well as in studies where changes in LSEC fenestration were linked to MASLD pathogenesis upon genetic interventions in LSECs^19,20,47^. Moreover, while Bondareva and colleagues observed ∼500 DEGs in LSECs after 3 months of WD-exposure, we found a similar amount of DEGs already after 4w, with largely similar functional annotation, again suggesting a role for LSECs in early MASLD^23^. Hammoutene and Rautou hypothesized that LSEC capillarization causes steatosis by hampering passive lipid transport and therefore inducing endogenous lipogenesis in hepatocytes^3^. Here, the WD-induced increase in capillarization indeed induced lipogenesis regulator *Srebf1*. However, the strongest capillarization occurred in the absence of endothelial ZEB2, a condition with decreased steatosis compared to *WT*. This suggests that additional phenomena determine the degree of steatosis induced by WD-feeding. We therefore propose to fine-tune the abovementioned hypothesis by stating that capillarization only partly determines the amount of steatosis. In our mouse model, we saw signs of capillarization through a prominent gene signature shift and basement membrane deposition while TEM revealed that fenestrae tended to be smaller and less numerous after 8w of endothelial ZEB2 loss and/or WD-exposure. One of the genes that based on the abovementioned genetic intervention studies in LSECs was proposed to be linked with decreased LSEC fenestration^20^i.e., *Pla2g4a*, was significantly upregulated upon the combined challenge with WD and *EC*^*Zeb2KO*^ (Supplementary Table 1). As the number of mice available for TEM analysis was limited, in order to obtain a more definitive picture on LSEC fenestration in our mouse model, complementary scanning electron microscopic analysis, including at later stages, is needed. We hypothesize that passive diffusion of lipids through the fenestrated LSECs may be complemented with active transport mechanisms and that the latter were amplified by endothelial ZEB2-loss (Fig. 7). In support, we found that endothelial ZEB2-loss in WD-exposed mice prominently activated PPARα-signaling, regulating lipid transport and metabolism, in LSECs. This implies that LSECs might have a ‘buffering’ role that protects against lipotoxicity in the parenchyma by actively taking up and metabolizing lipids. Such a role has been previously suggested for non-fenestrated ECs^48–50^. LSECs may be especially proficient in this task by expression of many receptors active in lipid transport^12,51,52^. Additional functional support for altered sinusoidal lipid transport and metabolism upon endothelial ZEB2-loss requires additional testing.

The majority of hepatocytic lipids in patients with MASLD originate from peripheral FAs^53,54^ and LSECs are capable of storing and degrading lipids following exposure to free FAs^55,56^. This Ppar(α)-driven role in lipid homeostasis in LSECs also implies that Ppar(α) agonists that have been proposed for MASLD treatment because of their role in hepatocytes^39^may offer additional benefit through their action on liver endothelium. In addition to its effect on EC metabolism, PPAR signaling has anti-inflammatory effects, also desired during treatment of MASH^39^. Like in other studies^57^the degree of inflammation and fibrosis was limited in our model, precluding us to evaluate whether the increased PPAR signaling upon endothelial ZEB2-loss protected against transition to MASH and fibrosis.

One of the prominent common responses of LSECs to ZEB2-loss and WD-exposure was cell proliferation (Fig. 7). Others, who interpret this response as a possible compensatory mechanism because of liver expansion, also reported proliferation of LSECs as response to WD^23,36^. While the increased proliferation tended to increase vascular branch density after 24w in mice on a SD, simultaneous hepatocyte hypertrophy in mice fed a WD overruled this increase, resulting in a significant decrease in vascular branch density per parenchymal area. While the number of vascular branches per hepatocyte was unaltered upon WD exposure, the hepatocyte hypertrophy likely requires an increased ratio to cope with higher energy and oxygen demands. Hence, during steatosis, the hepatic parenchyma is hypo-vascularized, potentially co-contributing to hepatocyte damage and representing a hypoxic trigger for angiogenesis seen during transition to MASH^58^. Whereas angiogenesis may drive the progression of MASLD from the transition to MASH onwards^24^, it may not directly promote steatosis. Indeed, blocking angiogenesis by antagonizing Angiopoietin-2 was shown to reduce hepatocyte ballooning and fibrosis, but did not affect steatosis^59^. In our previous studies, a more short-term ZEB2-loss in ECs caused expansion of the liver vasculature in the absence of increased proliferation, reflecting intussusceptive angiogenesis^7^. This indicates that genetic inactivation of endothelial *Zeb2* causes both proliferative and non-proliferative forms of angiogenesis. Altogether, angiogenesis is stage-dependently involved in MASLD progression.

We previously showed that endothelial ZEB2-loss both causes expression changes in LSECs as well as non-LSECs, mainly hepatic stellate cells^7^. Also in the brain and the heart, ZEB2-loss in neurons and cardiomyocytes, respectively, caused secondary expression changes in other cell-types^60,61^. Here, endothelial ZEB2-loss had a significant impact on communication among LSECs (Fig. 7). Interestingly, while PPARα signaling genes were among the most notable ones upregulated by combined WD and *Zeb2* knockout challenges, PPARα itself was also one of the important targets of LSEC-LSEC communication. Another intriguing ligand and target of LSEC-LSEC communication was vasoconstrictor *Edn1*, which was upregulated in response to 12 predicted ligand-receptor interactions (Fig. 5c; Supplementary Fig.S8). The most likely receptor on LSECs predicted for communication with LSECs through *Edn1* was endothelin receptor B (*Ednrb*; Supplementary Fig.S8), whose interaction in human umbilical vein ECs was shown to promote proliferation^62^. Increases in Endothelin-1 during steatosis have also been reported in a rat model, where its vasoconstrictive effect – most likely through interaction with its endothelin receptor A on hepatic stellate cells – was responsible for increased intrahepatic vascular resistance causing impaired blood flow^26,63^. The most prominent predicted target of the LSEC-LSEC communication was proliferating cell nuclear antigen (PCNA) clamp-associated factor (*Pclaf*; Supplementary Fig.S8), which has been associated with cell cycle progression in multiple tumors, including neuroblastoma, through its interaction with PCNA^64,65^. Hence, the increased LSEC proliferation may also be the result of altered LSEC-LSEC communication. Altogether, endothelial ZEB2-loss has an etiology-dependent effect on cell-cell communication in the liver.

Currently, weight loss is the main pragmatic and therapeutic option for patients with MASLD, but life-long compliance has proven to be problematic, invoking the need for drug development. Recently, a first anti-MASH drug (resmetirom) has been approved by the U.S. Food and Drug Administration and several phase 2/3 trials are ongoing (clinicaltrials.gov), including with aspirin and PPAR agonists^66^. While hepatocytes have been mostly the target of such drugs, LSECs are another highly appealing target given their multi-factorial involvement from early stages of MASLD onwards. To uncover which pathways are critical for the observed phenotypes, EC-specific expression modulation of individual factors, like PPARα, would be required. In addition, we conclude that endothelial ZEB2 has a stage-dependent effect on liver disease, which will necessitate customized approaches when considering ZEB2 as a target for therapy.

## Methods

### Mice and MASLD model

Mouse experiments were approved by the KU Leuven Animal Ethics Committee (Ethics Committee Dossier 169/2014, 208/2017 and 121/2019) and were performed in accordance with the Committee’s guidelines and those from directive 2010/63/EU of the European Parliament on the protection of animals used for scientific purposes and the ARRIVE guidelines. Mice were housed in filter top cages with wood bedding and cocoons as enrichment under standard conditions with 10/14-hour light/dark cycles. Mice had ad libitum access to SD (Rat/Mouse – Maintenance, Ssniff) or WD (high-fat/high sucrose/high cholesterol; TD.88137, Ssniff; the full composition of the diet is given in Supplementary Table S3) and water.

All interventions were done during the light cycle. Male mice (known to be more susceptible to diet-induced steatosis^67^) were used that were 5 weeks of age at the start of the experiment. Three different mouse lines were used: 1° tamoxifen-inducible EC-specific *Zeb2* knockout mice (*EC*^*Zeb2KO*^) generated by inter-crossing the tamoxifen-inducible *Cdh5-Cre*^*ERT2*^ driver line^68^ with *Zeb2*^*fl/fl*^ mice (on a 129 Sv/CD1 background) carrying a *Zeb2 exon 7* flanked by *loxP* sites^7,69^; 2° tamoxifen-treated *Cre*-negative littermates of *EC*^*Zeb2KO*^ mice (*EC*^*WT*^), used as *WT* control for phenotypic analysis^7^; 3° tamoxifen-inducible EC reporter mice newly generated for the current study by inter-crossing *Cdh5-Cre*^*ERT2*^ mice with mice carrying the RCE reporter (*EC*^*GFP*^)^33^used as *Cre-*controls for RNA profiling of sorted eGFP^+^ liver ECs, for comparative analysis of *Zeb2* expression in different organs and for part of the phenotypic analysis (Supplementary Fig.S1 + NoteS1). To induce efficient Cre-mediated recombination and *Zeb2* deletion in ECs, mice were intraperitoneally (i.p.) injected with 1 mg tamoxifen (Sigma) in 100 µL sunflower oil (Sigma) for 5 consecutive days (Supplementary Fig.S1). Because the sorted EC population used for expression profiling mainly (> 99%) represents LSECs, we consider that expression changes detected upon Cre-mediated *Zeb2* deletion largely reflect those in LSECs. We therefore, where appropriate, labeled the EC population in the methods, results and discussion sections and corresponding figures as ‘LSECs’ rather than ‘ECs’. Three weeks after the first tamoxifen injection, mice were randomized to SD or WD and were fed this diet for a period of 4w, 8w or 24w (Supplementary Fig.S1). Blood was drawn via the heart using a heparin-coated syringe and mice were euthanized by exsanguination under sodium pentobarbital (Dolethal^®^; 66.7 µg/g i.p.) anesthesia. Anesthesia depth was checked by toe pinch and, if necessary, mice received another i.p. injection with 22.2 µg/g sodium pentobarbital.

For immunohistological, weight and/or plasma analyses, mice were euthanized after 4w, 8w or 24w of diet under anesthesia as described above. Livers were subsequently perfusion-fixed with zinc-formalin (Sigma), followed by overnight immersion fixation with zinc-formalin. The left lateral lobe was processed for paraffin sectioning, the left medial lobe was kept overnight in 25% Tris-buffered saline (TBS)-sucrose, before embedding in TissueTek. Liver and WAT paraffin blocks were sectioned at 7 and 8 μm thickness, respectively. Liver cryosections were sectioned at 10 μm thickness. For cell isolation and gene profiling, weight, plasma and part of the immunohistological analyses, mice were euthanized under anesthesia (as described above) after 4w of diet and after a 4-hour starvation period, to reduce biological variation. The right medial and lateral lobe were dissected and immersion-fixed overnight with zinc-formalin before processing for paraffin- and cryo-sectioning as described above. The caudate lobe was isolated, snap-frozen and stored at -80 °C until further use for liver triglyceride determination and RNA isolation. For liver triglyceride determination, a lipid extraction was performed as described in de Haan et al.^70^ followed by applying a colorimetric triglyceride detection kit (MAK266, Sigma) according to the manufacturer’s instructions.

### Cell isolation and gene expression analysis

To study tissue-specific endothelial *Zeb2* expression, eGFP^+^ ECs were isolated from *EC*^*GFP*^ mouse livers, hearts, brains, visceral WAT, muscles, spleens, bone marrow and intestines and comparative gene expression was performed by qRT-PCR. To isolate LSECs, *EC*^*Zeb2KO*^ and *EC*^*GFP*^ (both Cre-positive) mice were perfused via the portal vein with 2 mL digestion buffer (1.2 U/mL dispase in Gey’s buffer). The liver was excised and incubated at 37 °C for 10 min followed by 10 min at room temperature in the same digestion buffer (see Supplementary Table 4). The suspension was shaken manually every 5 min, filtered through a 70 μm strainer and centrifuged for 5 min at 50 g to separate the hepatocytes from the non-parenchymal cell fraction. Hepatocytes were immediately snap-frozen after centrifugation and stored. To isolate LSECs from the non-parenchymal cell fraction, the hepatocyte-free suspension was used for Percoll density gradient centrifugation. After pelleting the cells, red blood cells were removed from the non-parenchymal cell fraction by incubation with erythrocyte lysis buffer (0.15 M NH_4_Cl; 10 mM KHCO_3_; 0.1 mM EDTA) for 5 min, followed by washing in phosphate-buffered saline (PBS). The resulting cell pellet was resuspended in fluorescence-activated cell sorting (FACS) buffer (2 mM EDTA; 25 mM HEPES pH7; 0.2% bovine serum albumin (BSA; in PBS) containing 7-aminoactinomycin D (7-AAD) as live/dead marker and filtered through a 40 μm mesh before cell sorting of 7AAD^−^/UV^−^/eGFP^+^ LSECs on a FACS Aria-III sorter.

To isolate other tissue-specific ECs, tissues were dissected, minced and digested in a customized digestion buffer (Supplementary Table 4). Heart, skeletal muscle, lung, kidney, adipose and spleen tissue digests were filtered through a 70 μm mesh in PBS containing 2% fetal bovine serum (FBS). After pelleting the cells for 7 min at 600 g, erythrocyte lysis was performed for 2 min before washing with PBS. Next, cells were resuspended in FACS buffer and filtered in a FACS tube. To isolate bone marrow ECs, bones were flushed with 10 mL Hank’s balanced salt solution using a 23–26 G syringe. Then, the cell suspension was processed like the tissue digests described above. To isolate brain ECs, the tissue homogenate was spun down in 2% FBS at 600 g for 7 min before resuspending the cells in DMEM with 20% BSA. After centrifuging at 600 g for 10 min, the pellet was resuspended in 0.05% trypsin at 37 °C for 2 min. Then PBS containing 2% FBS was added and the suspension was filtered through a 70 μm cell strainer. After pelleting the cells for 7 min at 600 g, erythrocyte lysis was performed for 2 min before washing with PBS. Next, cells were resuspended in FACS buffer and filtered through a 40 μm mesh before sorting. FACS data were analyzed with FACS DIVA software.

RNA was isolated from sorted ECs using Reliaprep (Promega) for RNA sequencing or qRT-PCR purposes. For RNA sequencing, RNA samples from *EC*^*GFP*^ and *EC*^*Zeb2KO*^ mice (*n* = 6 per diet and genotype) were quality-controled and prepared according to the Smart-seq2 method^71^. In brief, polyA + RNA was reverse transcribed using an oligo(dT) primer. Template switching by reverse transcriptase was achieved using an LNA-containing TSO oligo. The reverse transcribed cDNA was pre-amplified with primers for 18 cycles followed by clean-up. Tagmentation was performed on 500 pg of the pre-amplified cDNA with Tn5 followed by gap repair. The tagmented library was extended with Illumina adaptor sequences by PCR for 14 cycles and purified. The resulting sequencing library was measured on a Bioanalyzer and equimolar loaded onto a flow cell and sequenced according to the Illumina TruSeq v3 protocol on the HiSeq2500 with a single read 50 bp and dual 9 bp indices. Illumina adapter sequences and poly-A stretches were trimmed from the reads^72^. The remaining sequences were aligned to the mouse GRCm38 reference sequence using HISAT2 (version 2.1.0)^73^. Transcript abundance level (transcript count) was generated using HTSeq (version 0.9.1) based on the ENSEMBL 84 gene annotation^74^. The transcript counts were further processed using R software environment for statistical computing and graphics (version 3.4.0). Data normalization, removal of batch effect and other variance was performed using EDASeq R and RUVSeq packages^75,76^. Differential expression analysis was performed using edgeR (R package)^77^applying the negative binomial general linear model (GLM) approach. DEGs with false discovery rate (FDR) < 0.05, Benjamini-Hochberg multiple testing correction and expression level in *WT* samples of > 1 counts per million (CPM) were retained and used for further processing (i.e., principal component analysis, cluster analysis, Volcano plots, heatmaps, Gene Enrichment analysis by Toppfun (https://toppgene.cchmc.org/enrichment.jsp) and ligand-target prediction analysis by NicheNet (using an open source R implementation; https://github.com/saeyslab/nichenetr)^78^. The full code of the analysis is shown in a Supplementary NoteS2. To visualize overlap between DEG lists from each comparison, circos plots were generated using Metascape^79^. To analyze changes in predicted interactions between LSEC-derived ligands and targets in LSECs, V-Set And Immunoglobulin Domain Containing (Vsig)4^+^ Kupffer cells, hepatic stellate cells and hepatocytes from mice challenged with 24/36 weeks of WD, a Multi-NicheNet pipeline was applied on the scRNAseq data generated by Guilliams et al.^35^ (GSE192742). DEGs were determined based on a log(2)fold-change threshold of (-)0.25 and a *P*-value of less than 0.05. To integrate the CD45^+^ (including the Vsig4^+^ Kupffer cells) and CD45^−^ datasets, batch correction was performed in Multi-NicheNet, and selection of receptors relied solely on expression values and the top 100 interactions were extracted from the analysis. To obtain RNA from whole liver tissue, the liver was homogenized in TRIZol and RNA was isolated according to manufacturer’s instructions. For quantitative qRT-PCR, cDNA was made using the GoScript reverse transcription system (Promega) according to the manufacturer’s protocol. QRT-PCR was performed with the ABI system using Sybr green (ABI). *Gapdh* was used as reference gene after validation of its stability across the tested conditions through comparison with two other housekeeping genes (*Hprt1* and *Ywhaz*), as previously described^7^; for primer sequences, see Supplementary Table 4).

### Histological and Immunofluorescence staining and imaging

To assess liver fibrosis, paraffin sections were stained with Sirius red to reveal collagen. Hepatocyte sizes were determined by dividing tissue area by the manually counted number of hepatocytes (at least 800 per sample) on images of liver cryosections either stained with Rhodamine-Phalloidine (R415, ThermoFisher) to mark hepatocyte cell borders and Hoechst to label nuclei or with H&E. Hepatocyte sizes were corrected for polyploidy, as previously described^80,81^. Adipocyte sizes were measured by manually selecting a representative amount of cells on H&E-stained paraffin sections visualized by using a 40x objective on a brightfield microscope. Cell sizes and the amount of collagen were quantified using Image J. To evaluate inflammation throughout the MASLD model, paraffin sections were stained for CD45 and CD45-positive cells were counted in pericentral and periportal areas. To characterize LSEC capillarization and PPARα signaling target expression, immunofluorescence stainings were performed for collagen-type IV and FABP4, respectively. To assess LSEC proliferation, paraffin sections were double-stained for KI67 and eGFP. Antibodies were tested using a negative control (no primary antibody) and *WT* healthy mouse liver sections as positive control where we compare the expression pattern with stainings found on the manufacturers’ websites and in literature; for antibodies and concentrations see Supplementary Table 4. Where necessary, amplification was performed using a Cy3- or fluorescein-tyramide kit according to the manufacturer’s instructions (Perkin Elmer). Microscopy images were analyzed in Image J. To take brightfield or regular fluorescence microscopic images, a Leica upright brightfield or an upright Zeiss fluorescence microscope equipped with Axiovision imaging software were used, respectively. For confocal microscopy, a Zeiss confocal microscope was used (see Figure legends for more details).

### Vascular area analysis by quantitative vascular analysis tool

To determine branch density of the liver blood vasculature, paraffin sections were stained for ENDOGLIN. Sections were integrally imaged at high spatial resolution (i.e., 0.32175 μm/px) using a Nikon NiE-Marzhauser Slide Express 2 equipped with a Hamamatsu Orca Flash 4.0 camera (VIB-CBD). The vascular network of the entire sections was quantified in a tile-wise manner using the in-house developed Quantitative Vascular Analysis Tool (Q-VAT; Supplementary Fig.S11)^43^. The ENDOGLIN-stained fluorescent slide scanner images were pre-processed using the Q-VAT masking tool to generate a tissue mask for normalization and a binary vascular mask from the original immuno-stained images. These masks were partitioned into tiles with the same size as the original acquisition tiles and the masks were validated by overlaying them with the original immunofluorescence image. The following input parameters were used in the Q-VAT masking tool: Calibration, 0.32175 μm/px; Radius of biggest object, 16 μm; Particle size lower range, 1,000 μm²; Radius for median filtering, 15 μm and Thresholding method according to Huang et al.^82^. Automated analysis and quantification of the segmented vasculature was subsequently performed on each tile using Q-VAT with the following input parameters: Calibration, 0.32175 μm/px; Vascular compartment separation threshold, 10 μm; Close label radius, 3 μm; Prune ends threshold, 5 μm. The vessel diameter and branch density were calculated for each acquisition tile of the entire cross-sectional liver area. Tiles with aberrant values were excluded using a stationary cut-off, and outliers were removed using Robust regression and Outlier removal (ROUT) method with a ROUT coefficient Q = 1%. After outlier removal, the average vessel diameter and branch density were calculated for the entire cross-sectional area of the liver. Group-wise comparison was performed using two-way ANOVA with Tukey post-hoc test for multiple comparison. The Q-VAT tool can be downloaded, together with a more detailed user guide on GitHub (https://github.com/bramcal/Q-VAT.git).

### Published single-cell RNA sequencing dataset data-mining and re-analyses

The online portal from Kalucka et al.^34^ (https://endotheliomics.shinyapps.io/ec_atlas/; GSE99235 ref^83^; ArrayExpress: E-MTAB-8077) was used to evaluate inter-organ heterogeneity of adult mouse endothelial *Zeb2* expression. The online portal linked to Guilliams et al.^35^ (https://www.livercellatlas.org/datasets_diet.php; GSE192742) was used to evaluate heterogeneity in *Zeb2* expression in livers from lean and obese mice. *ZEB2* expression in healthy livers and livers from patients with MASLD was studied using the online portal from Guilliams et al.^35^ (https://www.livercellatlas.org/datasets_diet.php; GSE192742). The online portal described in Remmerie et al.^36^ (https://www.livercellatlas.org/datasets_diet.php; GSE156059) was used to evaluate *Zeb2* expression in liver cell clusters from mice challenged with SD or WD for 24w or 36w.

### TEM

TEM was performed to document the fenestration size and frequency, and calculate porosity of the liver sinusoids in vivo. Hereto, mice were perfusion-fixed with 2.5% glutaraldehyde in 0.05 M sodium-cacodylate buffer, pH 7.3. Liver slices were dissected, rinsed with 0.05 M Na-cacodylate buffer and post-fixed in 2% osmium tetroxide (OsO_4_) in sodium-cacodylate buffer at 4 °C for 24 h. Samples were dehydrated in a graded acetone series and embedded in Agar 100 Resin (Agar Scientific, Stansted, UK). Semi-thin (± 1 mm) sections were cut with a Reichert Jung Ultracut E microtome and stained with 0.1% thionin-0.1% methylene blue. The ultra-thin (± 70 nm) sections, on copper grids, were stained with uranyl acetate and lead citrate. Pictures were recorded on a JEOL JEM1400 (JEOL Europe BV, Zaventem, Belgium) transmission electron microscope (VIB BioImaging Core, platform Leuven). In order to be representative, analysis of fenestration involved a sufficient number of pictures, length of EC lining, and number of fenestrae (Supplementary Table 5).

### Plasma analyses

To evaluate liver function and systemic metabolic parameters, blood was drawn via the right heart using a heparin-coated 26G needle on a 1 mL syringe and stored as plasma at -80 °C after centrifuging at 3,000 rpm for 10 min. The plasma was sent to the UZ Leuven routine testing laboratory for determination of alanine transferase (ALT), aspartate transferase (AST), triglycerides and total cholesterol. VWF: Ag levels were determined on plasma on 0.37% sodium citrate as described in Brill et al.^84^. As a reference, a plasma pool of 20 untreated mice from our *Cdh5-Cre; Zeb2*^*fl/fl*^ mouse colony was generated.

### Statistics

Graphpad Prism 9 was used for all statistical analyses. Quantitative data are expressed as mean ± standard error of the mean (s.e.m). Normal distribution of the data (or the residuals in case of ANOVA) was tested in Graphpad Prism 9 after performing outlier detection. If not normally distributed, data were log-transformed before performing parametric statistical analysis. Student’s *t*-test was used to compare 2 groups. One-way or two-way ANOVA with Tukey post-hoc test was used to compare > 2 groups. *P* < 0.05 was considered statistically significant. Clear trends (0.05 < *P* < 0.2) are also indicated on graphs. In case of 4 group comparisons, statistical significance for the comparison between *EC*^*Zeb2KO*^ on SD *versus EC*^*WT*^ or *EC*^*GFP*^ on WD are systematically not indicated in the graphs as these comparisons were not considered as biologically relevant. Statistical analyses for the RNAseq data are described above.

## Electronic supplementary material

Below is the link to the electronic supplementary material.

Supplementary Material 1

Supplementary Material 2

## Data Availability

The RNA sequencing datasets generated for this study are available in the NCBI GEO repository (https://www.ncbi.nlm.nih.gov/geo/), under series number GSE224595. Any additional information required to reanalyze the data generated for and reported in this paper is available upon request from the corresponding author. All data was analyzed with standard programs and packages as indicated in the Methods.
