# Supplementary material for: Loss of endothelial ZEB2 in mice attenuates steatosis early during metabolic dysfunction-associated steatotic liver disease
Source: Sci Rep. 2025 Jul 2;15:23434. doi: 10.1038/s41598-025-05881-6 (PMC12222841; doi:10.1038/s41598-025-05881-6)
Supplement: Supplementary file 1 — Supplementary Material 1 [file 41598_2025_5881_MOESM1_ESM.pdf]

ONLINE SUPPLEMENT

Loss of endothelial ZEB2 in mice attenuates steatosis early during metabolic dysfunction-associated steatotic liver disease

Wouter Dheedene,[1] Stefaan Verhulst,[2] Louise Demuynck,[1] Bram Callewaert,[1] Willeke de Haan,[1] Stefan Vinckier,[3,4] Jore Van Wauwe,[1] Petra Vandervoort,[1] Marleen Lox,[1] Mathias Stroobants,[1] Renaud Lavend'homme,[1] Wilfred F.J. van IJcken,[5,6] Elizabeth A.V. Jones,[1,7] An Zwijsen,[1] Marc Jacquemin,[1] Leo van Grunsven,[2] Kimberly Martinod,[1] Danny Huylebroeck,[6,8] Eskeatnaf Mulugeta,[6,9] and Aernout Luttun[1]*

Affiliations: [1]Center for Molecular and Vascular Biology, Department of Cardiovascular Sciences, KU Leuven, Leuven, Belgium; [2]Liver Cell Biology research group, Vrije Universiteit Brussel, Brussels, Belgium; [3]Department of Oncology, Laboratory of Angiogenesis and Vascular Metabolism, KU Leuven, Leuven, Belgium; [4]Laboratory of Angiogenesis and Vascular Metabolism, Center for Cancer Biology, VIB, Leuven, Belgium; [5]Center for Biomics-Genomics, Erasmus University Medical Center, Rotterdam, The Netherlands; [6]Department of Cell Biology, Erasmus University Medical Center, Rotterdam, The Netherlands; [7] CARIM, Maastricht University, Maastricht, The Netherlands; [8]Department of Development and Regeneration, KU Leuven, Leuven, Belgium; [9]Department of Internal Medicine, Erasmus University Medical Center, Rotterdam, The Netherlands

*Corresponding author

A. Supplementary Figures

Supplementary Figure S1. Transgenic mouse models for EC-specific *Zeb2* expression manipulation and study design.

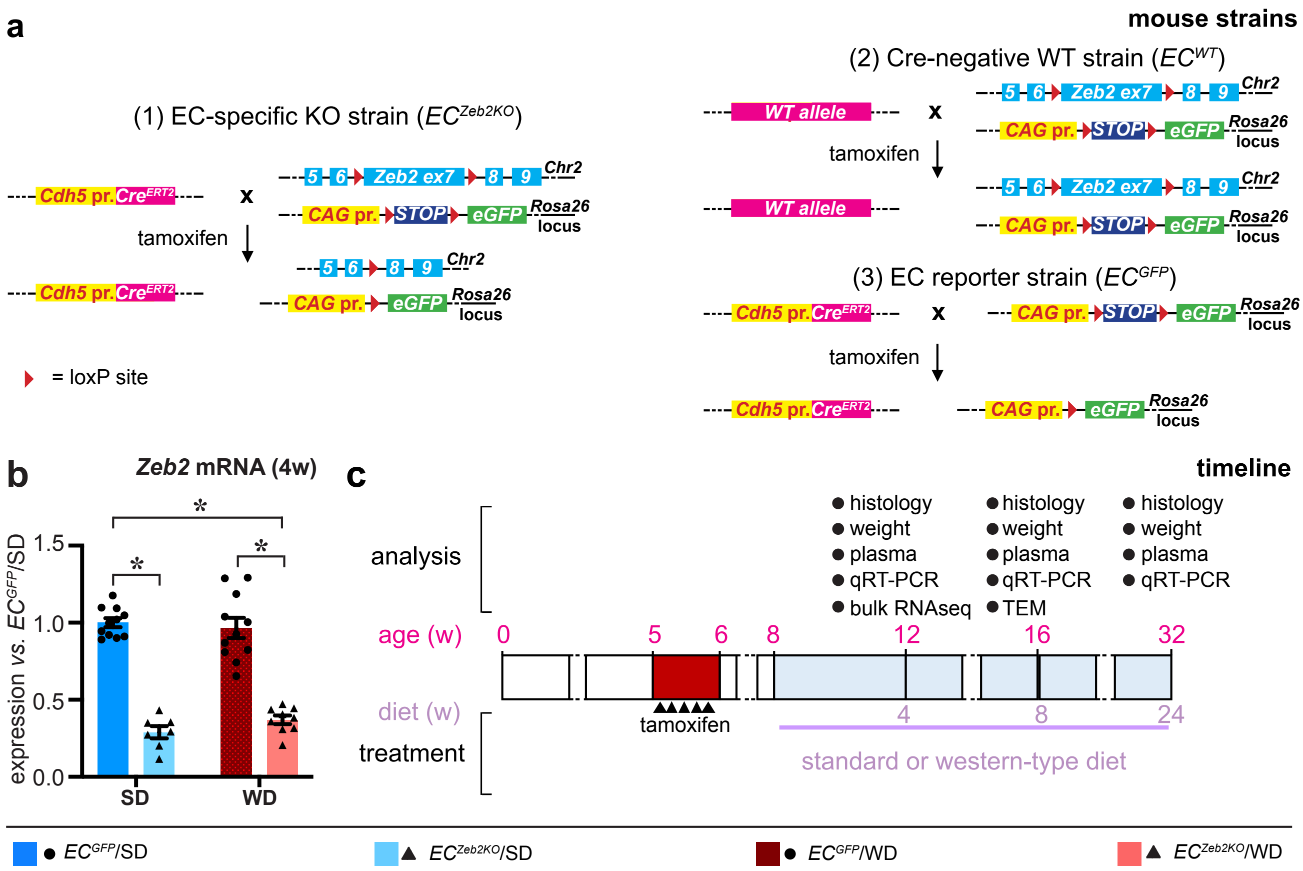

Supplementary Fig. S1 Transgenic mouse models for EC-specific *Zeb2* expression manipulation and study design. (a) Schematic diagrams showing the genetic strategy for EC-specific *Zeb2* knockout (EC^{Zeb2KO}; 1), EC-specific *Zeb2 wild-type* (EC^{WT}; 2) or EC-specific eGFP-based reporter mice (EC^{GFP}; 3). *Exon 7* encodes the last zinc finger of N-terminal zinc finger cluster, the Smad-binding domain, the homeodomain-like domain and the CtBP-interacting domain, and is the critical exon for intact Zeb2 functions[1]. eGFP: enhanced green fluorescent protein; pr.: promoter; Chr.: chromosome; CAG: CMV early enhancer/chicken beta-actin; *Rosa26* or *R26*: reverse orientation splice acceptor. (b) *Zeb2* mRNA expression in LSECs isolated by FACS from EC^{Zeb2KO} or EC^{GFP} livers after 4 weeks (w) of standard diet (SD) or western-type diet (WD; n=7-11). (c) Schematic diagram showing the study design for evaluating the role of ZEB2 during diet-induced metabolic dysfunction-associated steatotic liver disease. w: weeks; qRT-PCR: quantitative real-time polymerase chain reaction; RNAseq: RNA-sequencing; TEM: transmission electron microscopy. Quantitative data represent mean ± s.e.m. *: P<0.05 *vs.* indicated condition by two-way ANOVA with Tukey post-hoc test.

Supplementary Figure S2. Twenty-four weeks of western-type diet feeding induces advanced steatosis but not liver fibrosis and loss of endothelial ZEB2 does not affect inflammation or fibrosis.

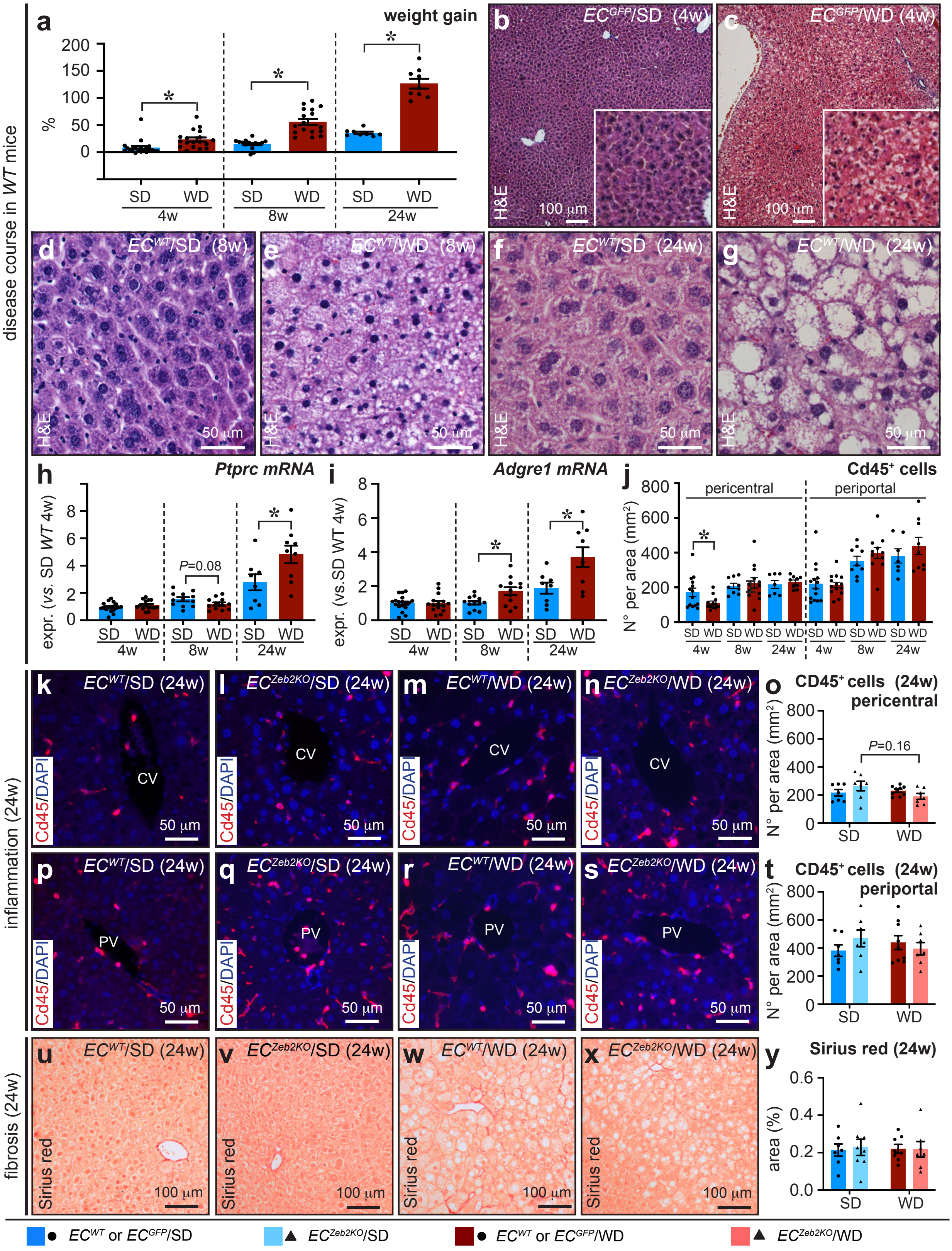

Supplementary Fig. S2 Twenty-four weeks of western-type diet feeding induces advanced steatosis but not liver inflammation or fibrosis, the latter two not affected by endothelial ZEB2-loss. (a) Bar graph showing weight gain of EC^{WT}/EC^{GFP} mice after 4 weeks (w; n=4 EC^{WT}/16 EC^{GFP} for SD; n=0 EC^{WT}/18

EC^{GFP} for WD), 8w (*n*=10 EC^{WT}/5 EC^{GFP} for SD; *n*=13 EC^{WT}/4 EC^{GFP} for WD) or 24w (*n*=3 EC^{WT}/5 EC^{GFP} for SD; *n*=4 EC^{WT}/5 EC^{GFP} for WD) of standard diet (SD) or western-type diet (WD) expressed as a percentage of the body weight at the start of the diet. (b,c) Hematoxylin and eosin (H&E)-stained liver cross-sections of EC^{GFP} mice after 4w of SD (*b*) or WD (*c*) and corresponding enlarged views in the insets. (d-g) H&E-stained liver cross-sections of EC^{WT} mice after 8w of SD (*d*) or 8w of WD (*e*) or 24w of SD (*f*) or 24w of WD (*g*). (h,i) mRNA expression of genes related to inflammation in whole livers from EC^{WT}/EC^{GFP} mice after 4w (*n*=4 EC^{WT}/14 EC^{GFP} for SD; *n*=0 EC^{WT}/15 EC^{GFP} for WD), 8w (*n*=9 EC^{WT}/3 EC^{GFP} for SD; *n*=0 EC^{WT}/12 EC^{GFP} for WD) or 24w (*n*=3 EC^{WT}/5 EC^{GFP} for SD; *n*=4 EC^{WT}/5 EC^{GFP} for WD) of SD or WD, normalized to the SD condition at 4w. (j) Quantification of the number of $CD45^{+}$ cells in pericentral (*left*) and periportal (*right*) areas in CD45-stained liver cross-sections from EC^{WT}/EC^{GFP} mice after 4w (*n*=0 EC^{WT}/14 EC^{GFP} for SD; *n*=0 EC^{WT}/12 EC^{GFP} for WD), 8w (*n*=9 EC^{WT}/0 EC^{GFP} for SD; *n*=12 EC^{WT}/0 EC^{GFP} for WD) or 24w (*n*=3 EC^{WT}/4 EC^{GFP} for SD; *n*=4 EC^{WT}/5 EC^{GFP} for WD) of SD or WD. (k-t) Representative images of CD45-stained (in red) liver cross-sections of the region around the central vein (CV; *k-n*) or portal vein (PV; *p-s*) from EC^{WT} (*k,m,p,r*) or EC^{Zeb2KO} (*l,n,q,s*) mice after 24w of SD (*k,l,p,q*) or WD (*m,n,r,s*) and corresponding quantification of the number of $CD45^{+}$ cells per area (*o,t*; *n*=3 EC^{WT}/4 EC^{GFP} for SD; *n*=4 EC^{WT}/5 EC^{GFP} for WD). DAPI (in blue) was used as nuclear stain. (u-y) Sirius red-stained liver cross-sections from EC^{WT} (*u,w*) or EC^{Zeb2KO} (*v,x*) mice after 24w of SD (*u,v*) or WD (*w,x*) and corresponding quantification of the $collagen^{+}$ area expressed as % of total tissue area (*y*; *n*=3 EC^{WT}/4 EC^{GFP} for SD; *n*=3 EC^{WT}/5 EC^{GFP} for WD). Quantitative data represent mean ± s.e.m; *: $P<0.05$ *vs.* indicated condition by Student's *t*-test. Pictures in *b,c* were taken with an EC Plan-Neofluar 5x/0.15 objective on a Zeiss Axio Imager Z1 equipped with an AxiocamMRc5 and Axiovision software. Pictures in *d-g* were taken with a PL-Fluotar 40x/0.70 objective on a Leica Leitz DMR BE microscope equipped with an AxiocamMRc5 and Axiovision software. Pictures in *k-n* and *p-s* were taken with an EC Plan-Neofluar 20x/0.50 M27 objective on a Zeiss Axio Imager Z1 equipped with an AxiocamMRc5 and Axiovision software. Pictures in *u-x* were taken with an PL-Fluotar 10x/0.30 PH1 objective on a Zeiss Axio Imager Z1 equipped with an AxiocamMRc5 and Axiovision software.

Supplementary Figure S3. Cell purity, knockout efficiency and quality control, segregation of sorted cell populations in pairwise comparisons of RNA sequencing data.

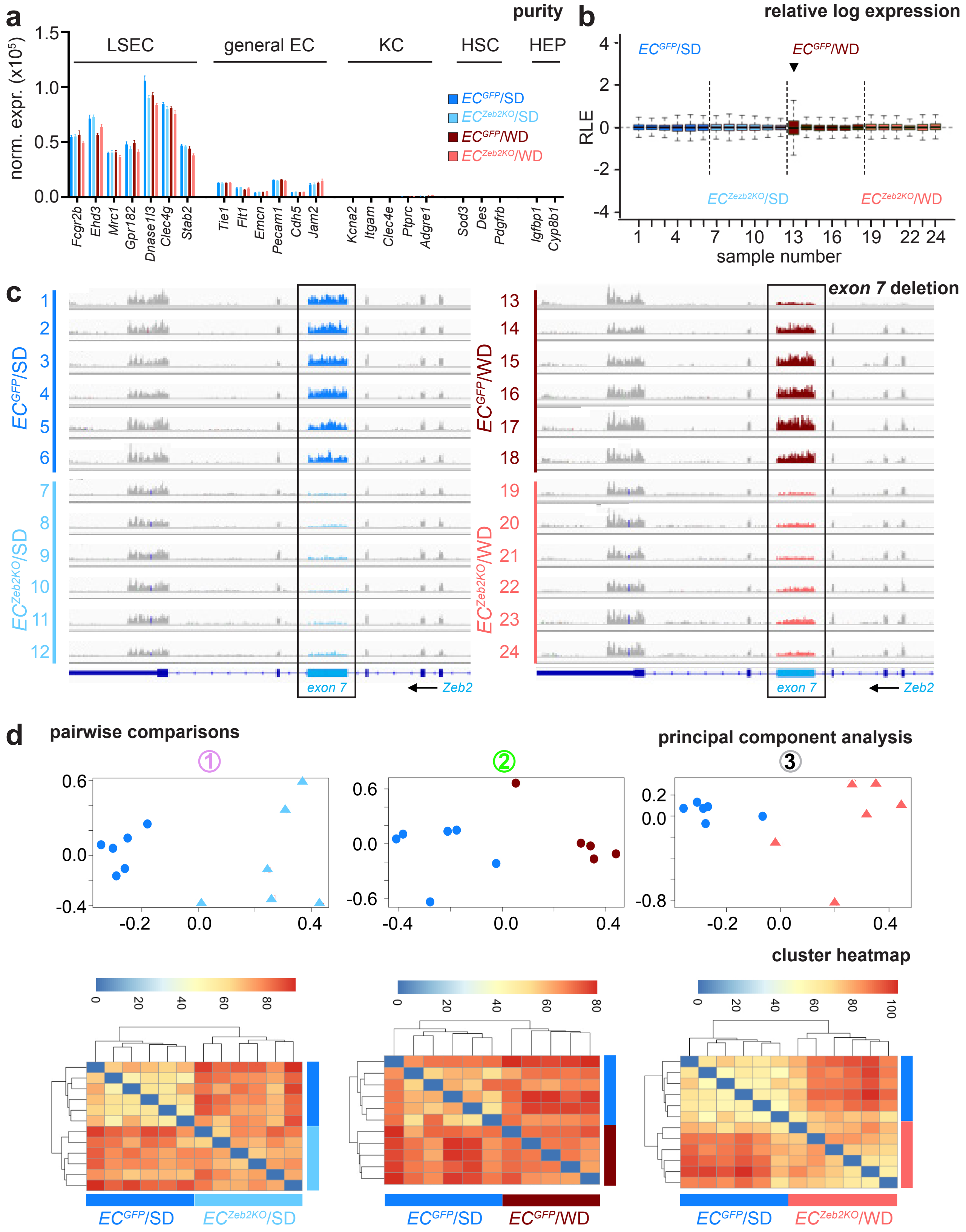

Supplementary Fig. S3 Cell purity, knockout efficiency and quality control, segregation of sorted cell populations in pairwise comparisons of RNA sequencing data. (a) Marker gene mRNA normalized expression level for different liver cell-types in all 4 conditions. (b) Relative log expression (RLE) plot

showing similar variation for all samples, except for sample 13 (indicated by black arrowhead). (c) Visualization of mapped reads on *chromosome 2,* in *Zeb2 exon 7* (black rectangle) and flanking regions using Integrated Genomics View (IGV) software. The RNA sequencing coverage is shown for each of the samples included in the study set-up (note the aberrant peaks in sample 13), revealing that *exon7* reads are exclusively diminished in LSECs in all mice from the EC^{Zeb2KO} conditions. (d) Principal component analysis plots (*top*) and cluster heatmaps sample distance (Euclidean distance) (*bottom*) of the 3 pairwise comparisons under study for all samples (except for sample 13). LSEC: liver sinusoidal endothelial cell; EC: endothelial cell; KC: Kupffer cell; HSC: hepatic stellate cell; HEP: hepatocyte; SD: standard diet; WD: western-type diet; KO: knockout.

Supplementary Figure S4. Volcano plots and heatmaps of differentially expressed genes from comparisons 1,2 and 3.

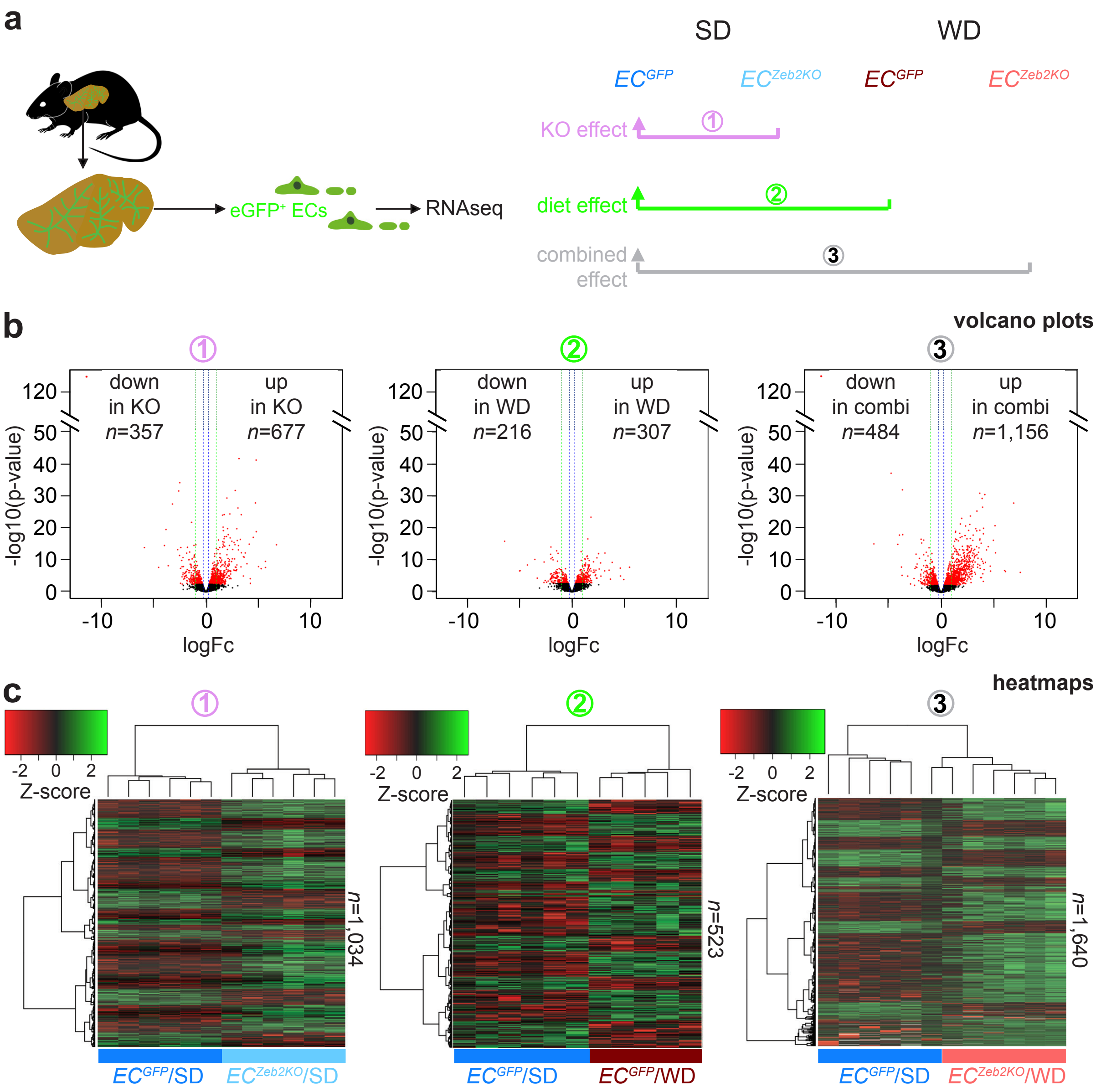

Supplementary Fig. S4 Comparisons 1-3 and their corresponding differentially expressed genes. (a) Schematic diagram of the RNAseq set-up (*left*) and overview of (3) pairwise comparisons (*right*). (b) Volcano plots showing genes up- or downregulated by the knockout (KO; comparison 1 in purple) or the diet (comparison 2 in green) or by the combined challenge ('combi'; comparison 3 in gray). (c) Heatmaps of the 3 pairwise comparisons under study. The number of differentially expressed genes is mentioned on the *right*, the color code for expression in shown *top left*.

Supplementary Figure S5. Functional annotation analysis related to Fig.3c, with separation of up- and downregulated genes.

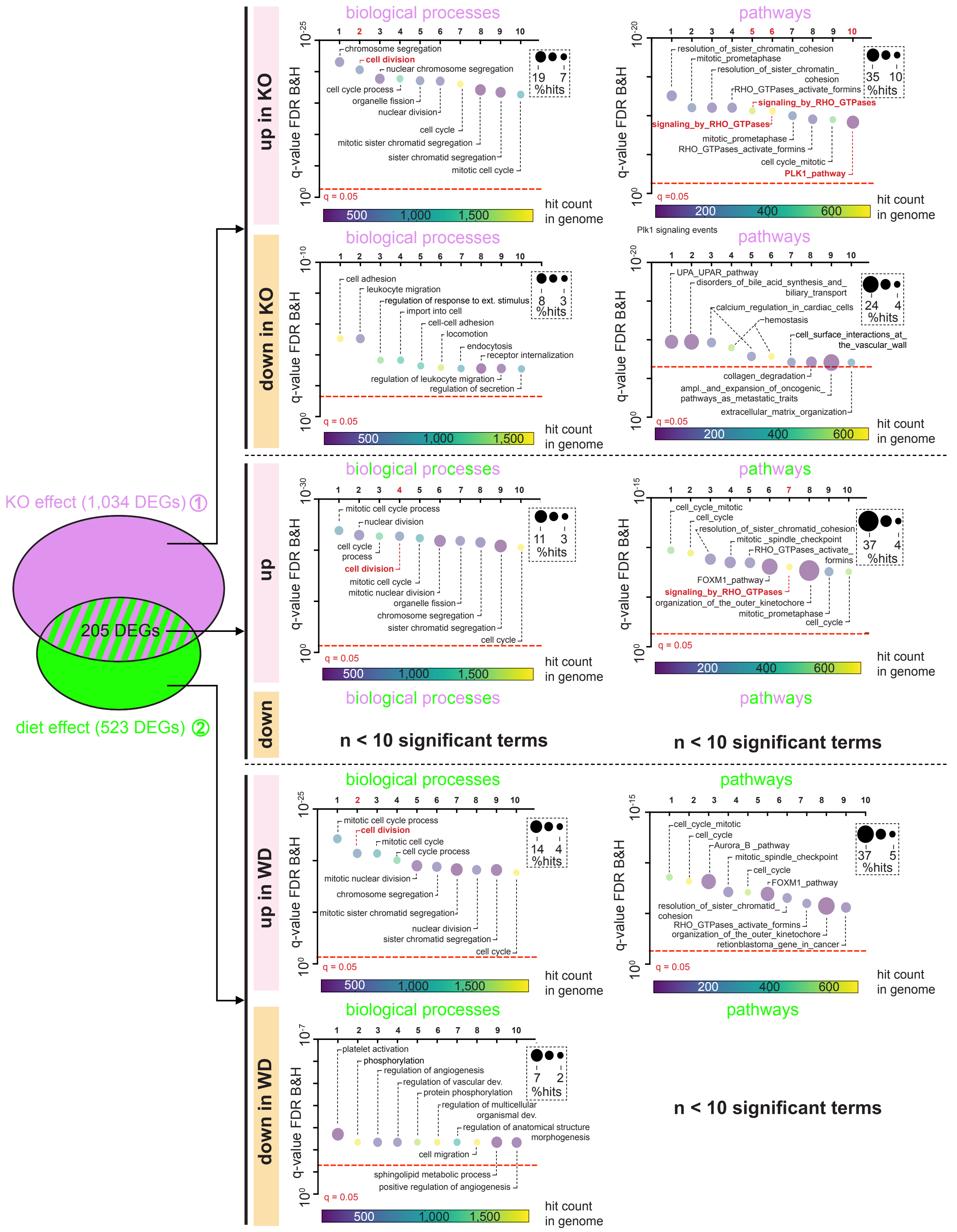

Supplementary Fig. S5 Functional annotation analysis on separated up- or downregulated genes (related to Fig.3c). Venn diagram (*left*) showing overlap (205 DEGs with expression changes in the same direction) between differentially expressed genes (DEGs) from the knockout (KO; comparison 1 in

purple) and the diet effect (comparison 2 in green). Bubble plots represent the top 10 functional terms (biological processes in the *middle*, pathways on the *right*) ranked according to false discovery rate (FDR) related to comparison 1 (*top*), comparison 2 (*bottom*) and the overlap between comparison 1 and 2 (*middle*), emerging from the functional annotation analysis performed separately on upregulated (pink bars) or downregulated (yellow bars) DEGs. Significance level indicated by red dotted lines. All quantitative data are also shown in Supplementary Table 1. Note that some analyses did not yield a sufficient number of significant terms.

Supplementary Figure S6. Functional annotation analysis related to Fig.4a, with separation of up- and downregulated genes.

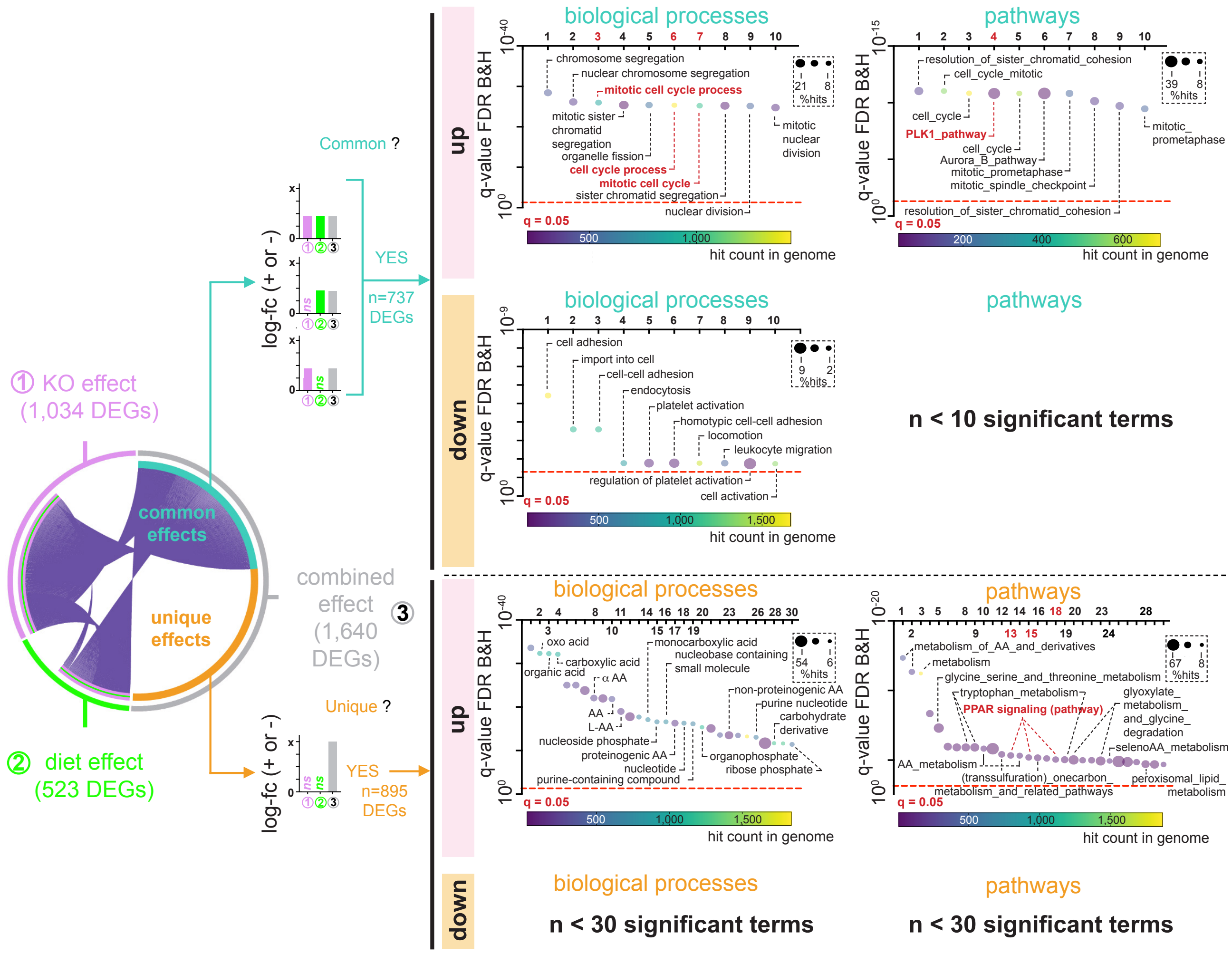

Supplementary Fig. S6 Functional annotation analysis on separated up- or downregulated genes (related to Fig.4a). Circos plot (*left*) showing overlap ('common', *i.e.*, genes with expression changes in the same direction; turquoise) or not ('unique'; orange) between DEGs from comparison 1 or 2 (single challenges: purple or green) and comparison 3 (combined challenge: gray). Schematic expression patterns for common or unique genes across single and combined challenges are shown next to the circos plot. Functional terms derived from the separated up- (pink bars) and downregulated (yellow bars) DEGs (full list: see Supplementary Table 1 for common (*top*) or unique (*bottom*). DEGs are plotted as bubble plots representing top 10 or 30 biological processes (*middle*) and pathways (*right*), respectively, ranked according to false discovery rate (FDR). Pathways of interest are highlighted in red. Significance level in bubble plots is indicated by red dotted lines. Note that some analyses did not yield a sufficient number of significant terms.

Supplementary Figure S7. Multi-NicheNet analysis reveals communication alterations in LSECs during MASLD.

a

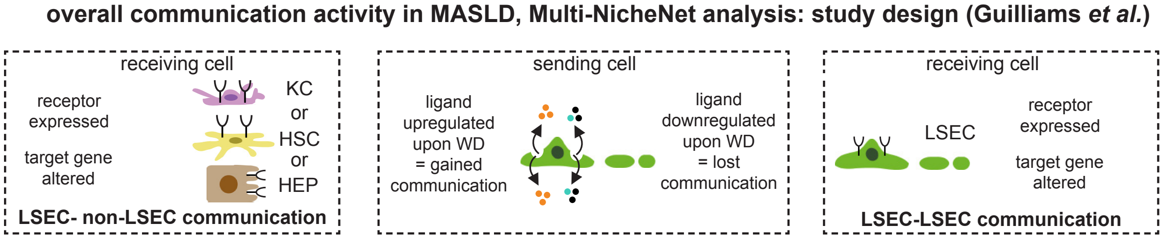

b

number of ligand-receptor pairs or predicted targets

ligand-receptor pairs	gained	lost	total
LSEC-LSEC	*n*=12	*n*=20	*n*=32
LSEC-KC	*n*=15	*n*=7	*n*=22
LSEC-HSC	*n*=17	*n*=6	*n*=23
LSEC-HEP	*n*=3	*n*=0	*n*=3

targets	gained		lost		total
	up	down	up	down	
LSEC-LSEC	*n*=27	*n*=20	*n*=42	*n*=32	*n*=121
LSEC-KC	*n*=61	*n*=31	*n*=18	*n*=35	*n*=145
LSEC-HSC	*n*=8	*n*=3	*n*=7	*n*=6	*n*=24
LSEC-HEP	*n*=20	*n*=6	*n*=0	*n*=0	*n*=26

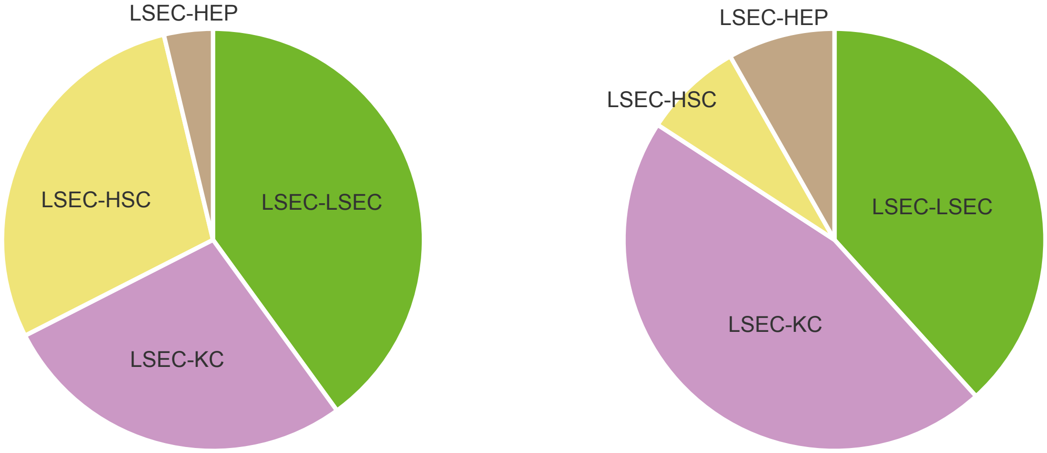

Supplementary Fig. S7 Multi-NicheNet analysis reveals communication alterations in LSECs during MASLD. (a) Schematic representation of the study design for Multi-NicheNet analysis of gained (‘G’) or lost (‘L’) communications of liver sinusoidal endothelial cells (LSECs) with other LSECs in green (*right*), or with non-LSECs (*i.e.*, Vsig4$^+$ Kupffer cells (KCs) in purple, hepatic stellate cells (HSCs) in yellow or hepatocytes (HEPs) in brown). (b) Numeric summary of the Multi-NicheNet output files extracted from Guilliams *et al.*[2] showing gained and lost communications through ligand-receptor pairs (*top left*) from LSECs to LSECs, KCs, HSCs or HEPs and correlated up- and down-regulated target genes (*top right*) in LSECs, KCs, HSCs and HEPs. Pie charts in the bottom show the total number of ligand receptor pairs (*left*) or associated targets (*right*) for each communication pair.

Supplementary Figure S8. EC^{Zeb2KO} and WD interact to alter LSEC-LSEC communication during MASLD.

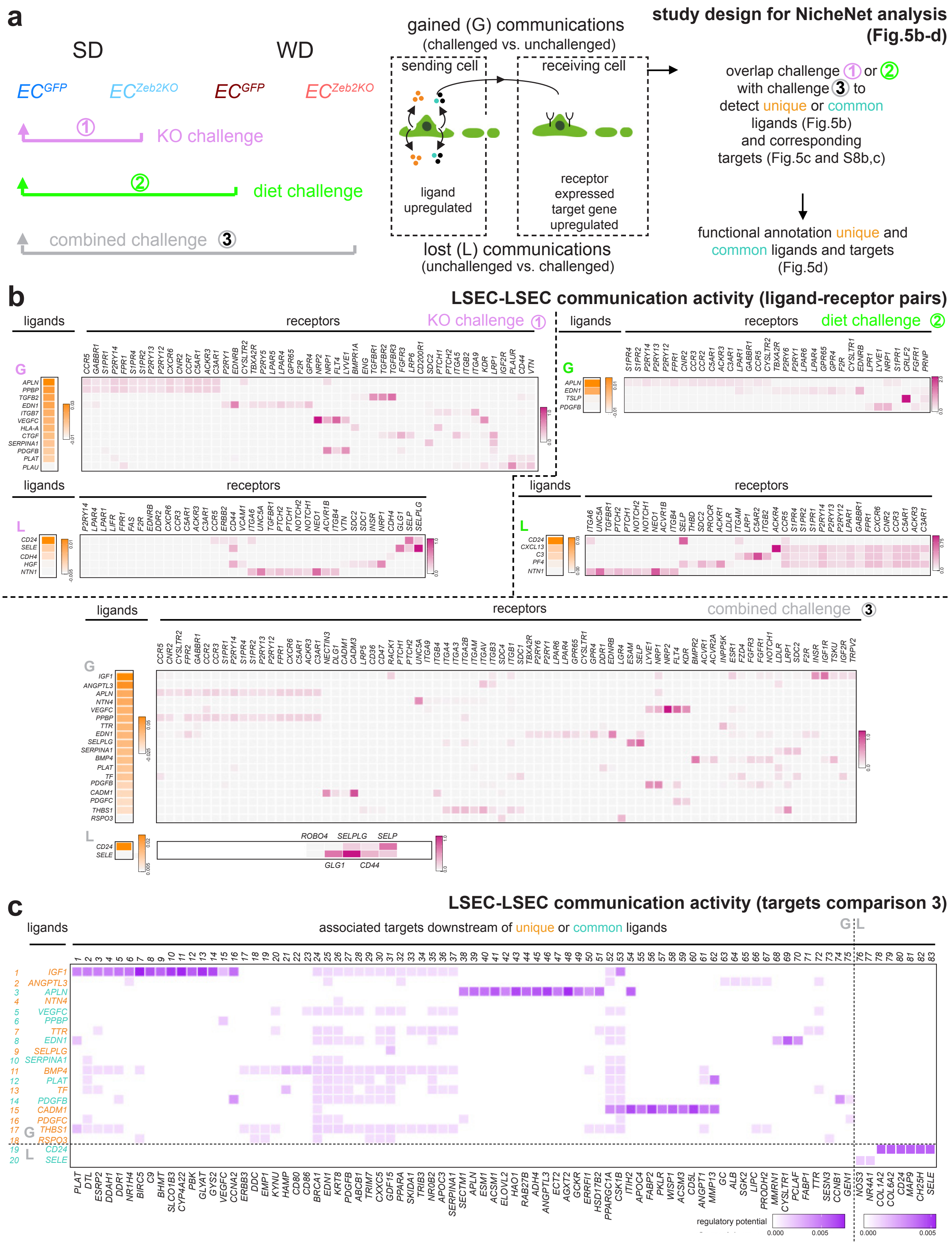

Supplementary Fig. S8 EC^{Zeb2KO} and WD interact to alter LSEC-LSEC communication during MASLD. (a) Schematic representation the study design for NicheNet analysis (*middle+right*) performed on the pairwise comparisons shown on the *left* both looking into LSEC-LSEC communications that are gained

(‘G’) *versus* those that are lost (‘L’) upon single knockout (KO; comparison 1), single diet (comparison 2) or combined challenge (comparison 3). (b) NicheNet output files showing heatmaps for both gained (‘G’; *top*) and lost (‘L’; *bottom*) communications of altered ligands in sending cells, and corresponding receptors expressed in receiver cells for comparison 1 (purple), 2 (green) and 3 (gray). (c) NicheNet output files showing a combined heatmap for gained (‘G’) and lost (‘L’) communications (separated by a dashed lines) of correspondingly altered target genes (*n*=83) in receiver cells for comparison 3 (gray). Functional annotation of the compiled list of ligands and associated targets (*n*=93) is shown in Fig.5d.

Supplementary Figure S9. Functional annotation analysis on separated gained or lost communication genes (related to Fig.5d).

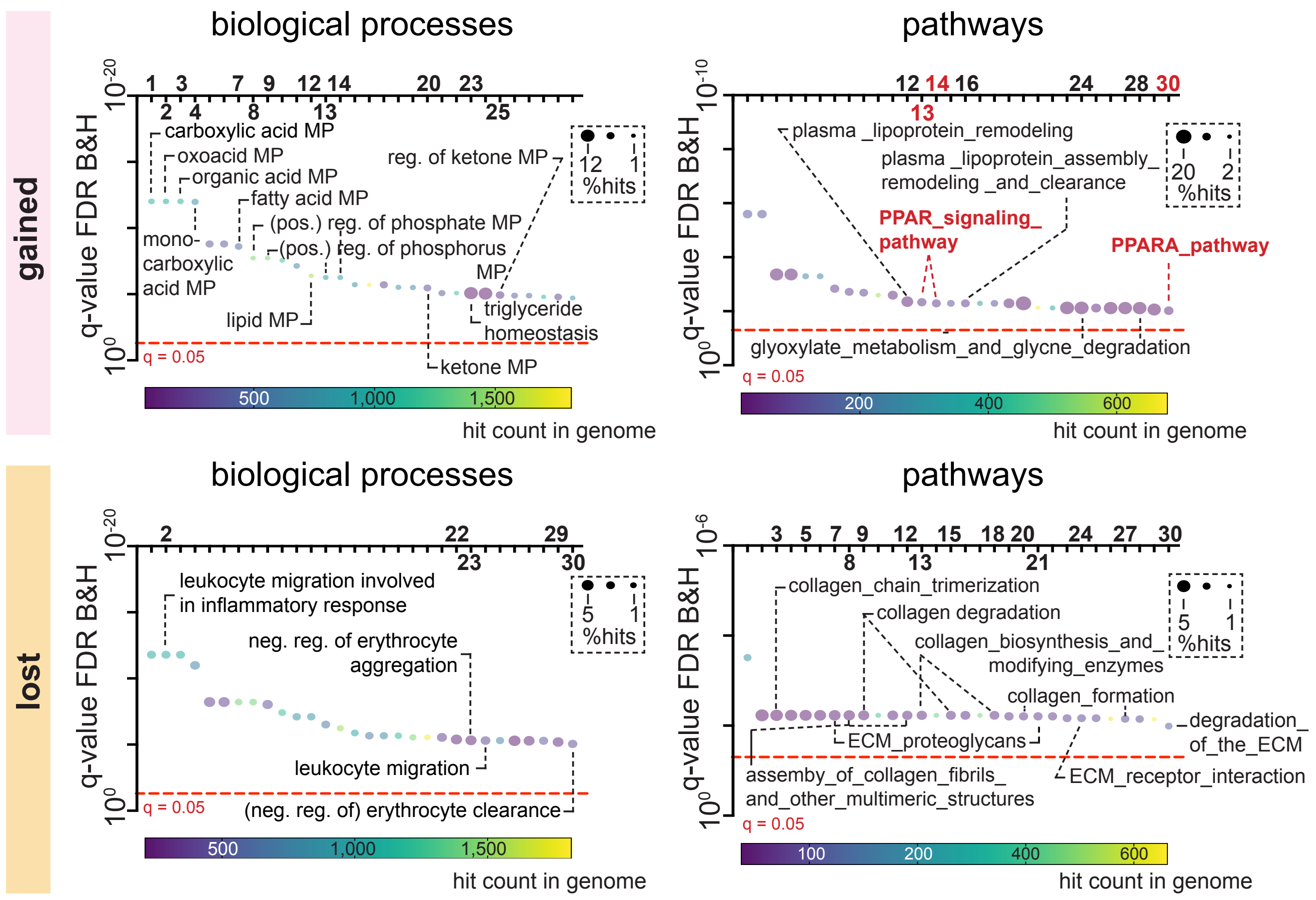

Supplementary Fig. S9 Functional annotation analysis on separated gained or lost communication genes (related to Fig.5d). Functional terms derived from the separated gained (pink bars) and lost (yellow bars) communication genes (full list: see Supplementary Table 2). DEGs are plotted as bubble plots representing the top 30 biological processes (*left*) and pathways (*right*) ranked according to false discovery rate (FDR). Pathways of interest are highlighted in red. Significance level in bubble plots is indicated by red dotted lines. MP: metabolic process.

Supplementary Figure S10. EC^{Zeb2KO} attenuates WD-induced steatosis development.

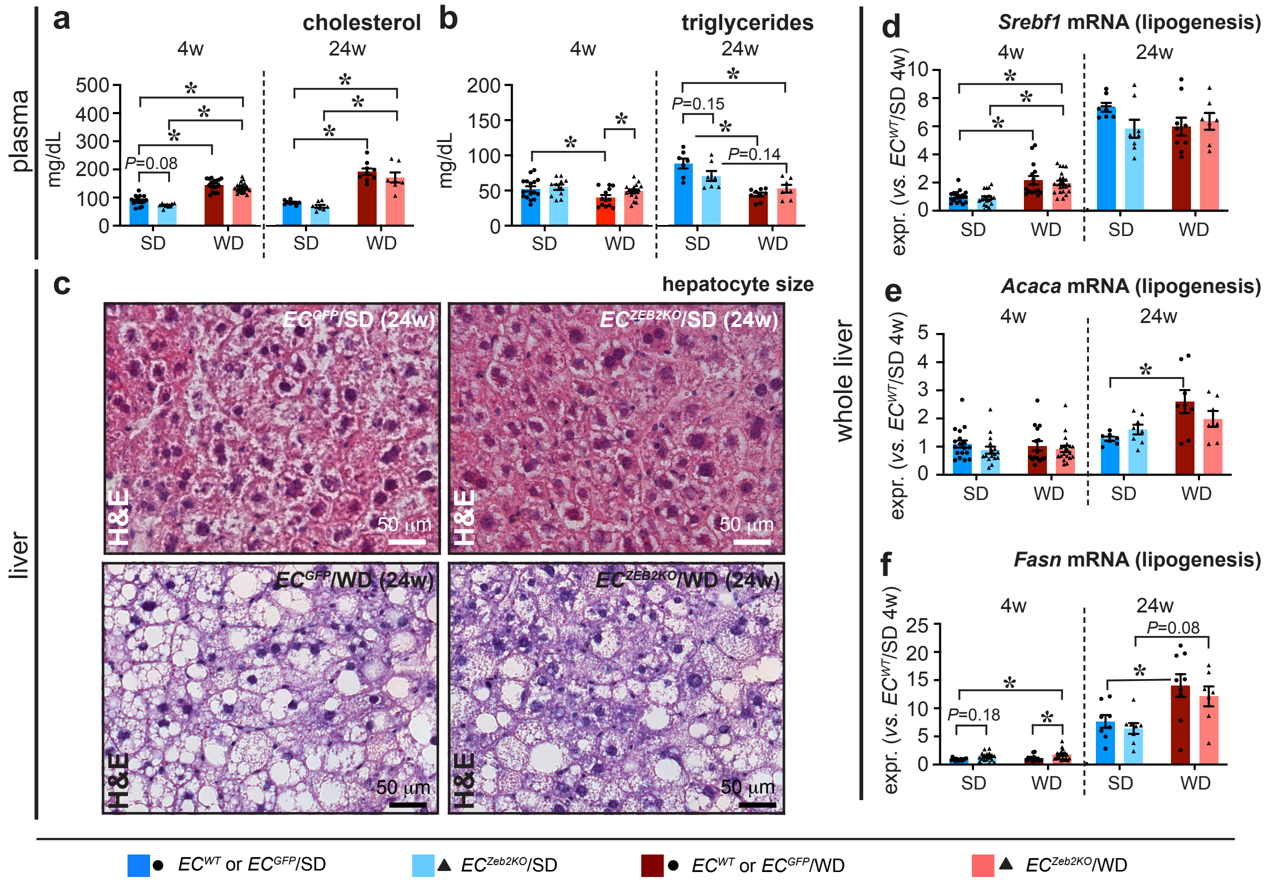

Supplementary Fig. S10 EC^{Zeb2KO} attenuates WD-induced steatosis development. (a,b) Quantification of cholesterol levels (*a*) and triglyceride levels (*b*) in plasma of mice from the indicated conditions after 4 weeks (w; *n*=0 EC^{WT}/16 EC^{GFP}/11 EC^{Zeb2KO} for SD; *n*=0 EC^{WT}/17 EC^{GFP}/20 EC^{Zeb2KO} for WD) or 24w (*n*=2 (*a*) or 3 (*b*) EC^{WT}/5 EC^{GFP}/8 (*a*) or 7 (*b*) EC^{Zeb2KO} for SD; *n*=4 EC^{WT}/5 EC^{GFP}/7 EC^{Zeb2KO} for WD) of standard (SD) or western-type diet (WD). (c) Representative images of H&E stained liver cross-sections from EC^{GFP} or EC^{Zeb2KO} mice after 24w of SD or WD feeding. (d-f) mRNA expression (expr.) of lipogenesis markers in whole livers from EC^{WT}/EC^{GFP} or EC^{Zeb2KO} mice after 4w (*n*=2 (*f*) or 4 (*d,e*) EC^{WT}/13 (*d*) or 14 (*e,f*) EC^{GFP}/16 (*e*) or 17 (*d,f*) EC^{Zeb2KO} for SD; *n*=0 EC^{WT}/14 (*f*) or 15 (*d,e*) EC^{GFP}/19 (*e*) or 21 (*f*) or 22 (*d*) EC^{Zeb2KO} for WD) and 24w (*n*=2 (*e*) or 4 (*d,f*) EC^{WT}/5 EC^{GFP}/8 (*d,e*) or 9 (*f*) EC^{Zeb2KO} for SD; *n*=4 EC^{WT}/4 (*e*) or 5 (*d,f*) EC^{GFP}/7 EC^{Zeb2KO} for WD) of SD or WD. All qRT-PCR data are normalized to the EC^{WT}/SD 4w condition. Quantitative data represent mean ± s.e.m; *: P<0.05 *vs.* indicated condition by two-way ANOVA with Tukey post-hoc test. Pictures in *c* were taken with an EC Plan-Neofluar 20x/0.50 M27 objective on a Zeiss Axio Imager Z1 equipped with an AxiocamMRc5 and Axiovision software.

Supplementary Figure S11. Q-VAT work flow.

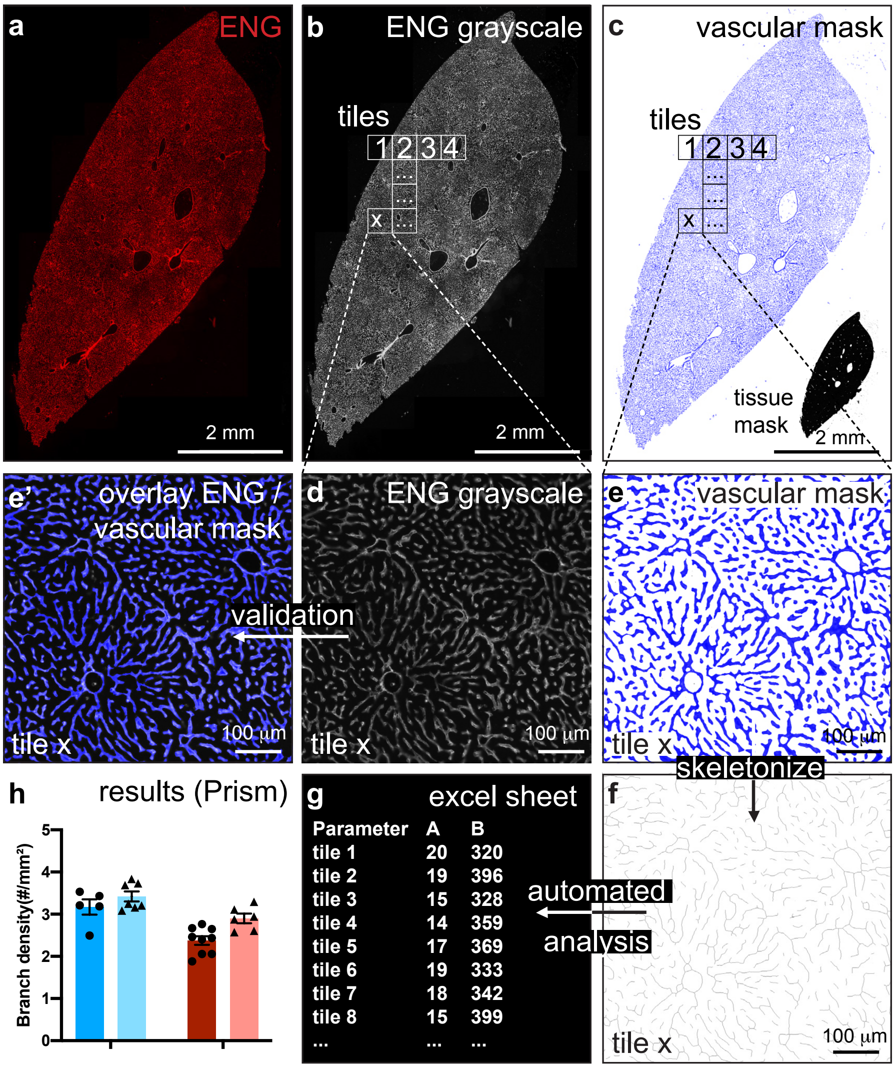

Supplementary Fig. S11 Q-VAT work flow. (a-c) Cross-section of a mouse liver stained for endoglin (ENG) in red recorded by a slide scanner (*a*), the corresponding grayscale image (*b*) and vascular mask generated by Q-VAT (in blue; *c*). The corresponding tissue mask generated by Q-VAT software is shown as an inset in the *lower-right* in *c*. After scanning the entire liver cross-section, the image is divided in tiles (d,e) Before tiles are analyzed, a validation step is included in which the appropriate overlay between the immunofluorescence image and the generated vascular mask is verified (*e'*). Next, for each validated tile the software will analyze several parameters, some of which require additional processing of the vascular mask (*e.g.*, generation of a skeletonized mask for measuring branching density; (f). All parameters are logged in an excel sheet for each tile. (g) After averaging the data across all tiles from liver, the data are plotted in Graphpad prism (h). Pictures in *a-c* were taken with a Plan Apo 20x (NA 0.75) objective on a Nikon Eclipse Ni-E with Marzhauser Slide Express 2 equipped with a Hamatsu Orca Flash 4.0 camera and NIS Elements software.

Supplementary Figure S12. Adipocyte hypertrophy.

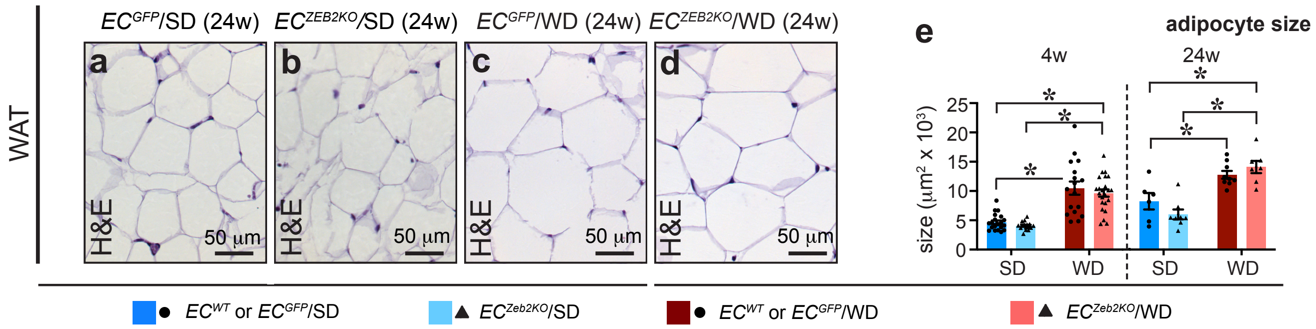

Supplementary Fig. S12 Endothelial ZEB2 loss does not affect diet-induced adipocyte hypertrophy. (a-d) Representative images of H&E stained cross-sections of visceral white adipose tissue (WAT) from EC^{WT}/EC^{GFP} (*a,c*) or EC^{Zeb2KO} (*b,d*) mice after 4w of SD (*a,b*) or WD (*c,d*) feeding and corresponding quantification (*e*) of adipocyte size after 4w (*n*=4 EC^{WT}/16 EC^{GFP}/16 EC^{Zeb2KO} for SD; *n*=0 EC^{WT}/17 EC^{GFP}/23 EC^{Zeb2KO} for WD) and 24w (*n*=2 EC^{WT}/4 EC^{GFP}/8 EC^{Zeb2KO} *for* SD; n=4 EC^{WT}/5 EC^{GFP}/7 EC^{Zeb2KO} *for* WD) of SD or WD-feeding. Quantitative data represent mean ± s.e.m; *: P<0.05 *vs.* indicated condition by two-way ANOVA with Tukey post-hoc test. Pictures in *a-d* were taken with an EC Plan-Neofluar 20x/0.50 M27 objective on a Zeiss Axio Imager Z1 equipped with an AxiocamMRc5 and Axiovision software.

B. Supplementary Tables

Supplementary Table 1: related to Fig. 3, Fig.4, Supplementary Fig.S5 and Supplementary Fig.S6: see additional excel file 'Supplementary tables 1 and 2'.

Supplementary Table 1.1. (a) Differentially expressed gene (DEG) list comparison 1, 2 and 3. (b) DEG list overlap comparison 1 and 2. (c) DEG list overlap comparison 1 or 2 and 3. (d) DEG list unique for comparison 3. Data in *a* relate to Fig. 3a. Data in *b* relate to Fig. 3b. Data in *c,d* relate to Fig. 4a. 'ns' in *C* means not significantly altered. *P*-values in *a-d* are corrected *P*-values for multiple comparisons.

Supplementary Table 1.2. (a) Functional annotation analysis on the combined up- or downregulated DEGs of comparison 1, 2 (biological processes + pathways). (b) Functional annotation analysis of overlap comparison 1 and 2 (biological processes + pathways). (c) Functional annotation analysis of overlap comparison 1 or 2 and 3 (biological processes + pathways). (d) Functional annotation analysis of unique genes for comparison 3 (biological processes + pathways). Numbers in the top row (… from …) refer to the number of output genes recognized by ToppGene from the number of input genes. FDR B&H: false discovery rate according to Benjamini-Hochberg. Data in *a,b* relate to Fig. 3c. Numbering in column A corresponds to numbering on the X-axis in Fig. 3c. Data in *c,d* relate to Fig. 4a. Numbering in columns W and AE corresponds to numbering on the X-axis in Fig. 4a.

Supplementary Table 1.3. (a) Functional annotation analysis on the separated up- or downregulated DEGs of comparison 1, 2 (biological processes + pathways). (b) Functional annotation analysis of overlap comparison 1 and 2 (biological processes + pathways). (c) Functional annotation analysis of overlap comparison 1 or 2 and 3 (biological processes + pathways). (d) Functional annotation analysis of unique genes for comparison 3 (biological processes + pathways). Numbers in the top row (… from …) refer to the number of output genes recognized by ToppGene from the number of input genes. FDR B&H: false discovery rate according to Benjamini-Hochberg. Data in *a,b* relate to Supplementary FigS5. Numbering in column A corresponds to numbering on the X-axis in Supplementary FigS5. Data in *c,d* relate to Supplementary Fig.S6. Numbering in columns Y and AJ corresponds to numbering on the X-axis in Supplementary Fig.S6.

Supplementary Table 1.4. Differentially expressed genes (DEGs) related to PPAR signaling.

Supplementary Table 2: related to Fig. 5 and Supplementary Fig.S9: see additional excel file 'Supplementary tables 1 and 2'.

Supplementary Table 2.1. (a) Secreted gene subset in comparison 1, 2, 3. (b) Common secreted gene subset comparison 1 or 2 and 3. (c) Unique secreted gene subset comparison 3. *P*-values in *a-c* are corrected *P*-values for multiple comparisons. Data in *a-c* relate to Fig. 5a.

Supplementary Table 2.2. Functional annotation analysis of combined gain and lost communication genes (ligands and associated targets; biological processes + pathways) from comparison 3. Numbers in the top row (… from …) refer to the number of output genes recognized by ToppGene from the number of input genes. FDR B&H: false discovery rate according to Benjamini-Hochberg. Data relate to Fig. 5d. Numbering in column A corresponds to numbering on the X-axis in Fig. 5d.

Supplementary Table 2.3. Functional annotation analysis of separated gain and lost communication genes (ligands and associated targets; biological processes + pathways) from comparison 3. Numbers in the top row (… from …) refer to the number of output genes recognized by ToppGene from the number of input genes. FDR B&H: false discovery rate according to Benjamini-Hochberg. Data relate to Supplementary Fig.S9. Numbering in column A corresponds to numbering on the X-axis in Supplementary Fig.S9.

Supplementary Table 3: Western-type diet composition

**Component**	**g/kg**	**remarks**
Casein	195.0	
DL-Methionine	3.0	
Sucrose	341.46	34% (by weight)
Corn starch	150.0	
Anhydrous Milkfat	210.0	
Cholesterol	1.5	0.2% total cholesterol
Cellulose	50.0	
Mineral Mix (AIN-76)	35.0	sodium chloride (193.7 g/kg); potassium citrate, monohydrate (576.0 g/kg); potassium, sulfate (136.1 g/kg); magnesium oxide (62.8 g/kg); manganous carbonate (9.2 g/kg); ferric citrate (15.7 g/kg); zinc carbonate (4.2 g/kg); cupric carbonate (0.8 g/kg); potassium iodate (0.026 g/kg); sodium selenite, pentahydrate (0.026 g/kg); chromium potassium sulfate, dodecahydrate (1.4 g/kg)
Calcium carbonate	4.0	
Vitamin Mix (Teklad 40060)	10.0	p-aminobenzoic acid (11.0 g/kg); ascorbic acid, coated, 97.5% (101.7 g/kg); biotin (0.044 g/kg); vitamin B12, 0.1% in mannitol (2.97 g/kg); calcium pantothenate (6.6 g/kg); choline dihydrogen citrate (349.7 g/kg); folic acid (0.2 g/kg); inositol (11.0 g/kg); vitamin K3, menadione (4.95 g/kg); niacin (9.91 g/kg); pyridoxin hydrochloride (2.2 g/kg); riboflavin (2.2 g/kg); thiamin (2.2 g/kg); vitamin A palmitate, 500,000IU/g (3.961 g/kg); vitamin D3, cholecalciferol, 500,000 IU/g (0.44 g/kg); vitamin E, DL-alpha tocopheryl acetate, 500 IU/g (24.2 g/kg); corn starch (466.7 g/kg)
Ethoxyquin	0.04	
**FA type**	**% of diet**	**Standard deviation**
Total	20.7	1.5
Saturated	12.8	0.8
Monounsaturated	5.6	0.5
Polyunsaturated	1.0	0.2
Unknown	1.3	0.3

Supplementary Table 4a. Digestion conditions for different organs

Tissue	Enzyme	Concentration	DNAse (U/mL)	Temp (°C)	Time (min.)
liver	dispase	1.2 U/mL	0	37-20	10-10
brain	collagenase	0.7 mg/mL	5	37	90
heart	collagenase	1.5 mg/mL	0	20	45
muscle	collagenase	1.5 mg/mL	0	37	45
lung	collagenase	1.5 mg/mL	0	37	45
kidney	collagenase	0.7 mg/mL	0	20	45
intestine	collagenase	1.5 mg/ml	5	37	60
adipose	collagenase	0.7 mg/mL	0	20	45
spleen	collagenase	0.7 mg/mL	5	20	45

Supplementary Table 4b. Primer sequences for qRT-PCR

Mouse gene	Forward primer (5‘ > 3’)	Reverse primer (5’ > 3’)
Acaca	*accgccagcttaaggacaac*	*tggggatgttccctctgtttg*
Adgre1	*ggacactgtgggttctgagg*	*caggagcagcccaagatgaa*
Cd34	*cagttggggaagtctgtggt*	*tccccatcagttcctaccaa*
Fasn	*gctcagcatggtcgcttctt*	*agatagccatgcccagaggg*
Gapdh	*ccgcatcttcttgtgcagt*	*gaatttgccgtgagtggagt*
Ptprc	*gccccgggatgagacagt*	*tttgaaagcccgagtgcct*
Srebf1	*aagtgcacacaaaagcaaatcact*	*acttcgggtttcatgccctc*
Zeb2	*ggactgcaagacggaagaca*	*ttgagtcggtggtcaagctc*

Supplementary Table 4c. Antibodies/reagents for immunofluorescence staining and cell isolation

Target	Ab species	conjugate	use, dilution	Antigen retrieval	Catalog number
KI67	rabbit	-	IF, 1:1600	Tris EDTA pH9	Ab15580
(e)GFP	chicken	-	IF, 1:100	Tris EDTA pH9; Dako target retrieval solution pH 6	Ab13970
ENDOGLIN	goat	-	IF, 1:100	Dako target retrieval solution pH 6	RD AF1320
VE-CADHERIN	goat	-	IF, 1:50	Dako target retrieval solution pH 6	RD AF1002
FABP4	goat	-	IF, 1:100	Tris EDTA pH9	RD AF1443
Phalloidin	-	Rhodamine	IF, 1:250	-	ThermoFisher R415
Collagen-type IV	rabbit	-	IF, 1:200	Dako target retrieval solution	Biorad 2150-1470

				pH 6	
CD45	Rat	-	IF, 1:100	Dako target retrieval solution pH 6	BD 533081
anti-Chicken IgG	donkey	Alexa-488	IF, 1:100	-	Jackson 703-546-155
anti-Rabbit IgG	goat	biotin	IF, 1:300	-	Dako E0432
anti-Goat IgG	rabbit	biotin	IF, 1:300	-	Dako E0466
anti-Rat IgG	rabbit	biotin	IF, 1:500	-	Invitrogen A18919
anti-Rabbit IgG	goat	Alexa-568	IF, 1:200	-	Invitrogen A11036
anti-Chicken IgG	goat	AF555	IF, 1:1500	-	Invitrogen A21437
anti-Rabbit IgG	goat	Alexa-488	IF, 1:1500	-	Invitrogen A11088

Supplementary Table 5: Fenestration analysis on TEM

	SD/EC^{WT}	SD/EC^{ZEB2KO}	WD/EC^{WT}	WD/EC^{ZEB2KO}
Number of biological replicates	5	2	3	4
	average ± s.e.m.			
Number of TEM pictures/replicate	5 ± 2	8 ± 4	7 ± 3	7 ± 3
Number of fenestrae/replicate	25 ± 21	27 ± 9	27 ± 25	35 ± 16
EC length (µm)/replicate	46 ± 21	79 ± 17	48 ± 32	85 ± 22
	MIN-MAX			
Number of TEM pictures/replicate	4-9	4-11	4-10	4-10
Total number of fenestrae/replicate	5-44	18-36	8-60	13-49
Total EC length (µm)/replicate	19-62	62-96	22-91	59-106

C. Supplementary notes

Supplementary Note S1. *The two mouse strains used as wild-type controls behave similarly upon exposure to standard or western-type diet.*

For the current study, in addition to the EC^{WT} control (which corresponds to the EC^{Zeb2KO} strain but without the *Cdh5-Cre*^{ERT2} allele; **Supplementary Fig. S1**), we also generated an EC^{GFP} control strain (which does not carry *floxed Zeb2 exon 7* alleles, but a recombination readout cassette; **Supplementary Fig. S1**). In order to increase statistical power, for some parameters we pooled both strains for the *wild-type* condition. Here, we show that pooling is appropriate since both strains behave similarly upon feeding the standard or western-type diet. For datasets where both *wild-type* strains were pooled, the number of mice for each strain is mentioned in the corresponding figure legend.

	strain	*n*=…	SD	*P*=…	strain	*n*=…	WD	*P*=…
AST (U/mL; 24w; plasma)	EC^{GFP}	5	39 ± 6	-	EC^{GFP}	5	186 ± 39	ns
	EC^{WT}	2	41 ± 6		EC^{WT}	4	181 ± 35	
ALT (U/mL; 24w; plasma)	EC^{GFP}	5	28 ± 5	ns	EC^{GFP}	5	218 ± 36	ns
	EC^{WT}	3	23 ± 3		EC^{WT}	4	305 ± 103	
cholesterol (mg/dL; 24w; plasma)	EC^{GFP}	5	83 ± 5	ns	EC^{GFP}	5	195 ± 11	ns
	EC^{WT}	3	81 ± 2		EC^{WT}	4	188 ± 25	
Body weight gain (%; 24w)	EC^{GFP}	5	36 ± 4	ns	EC^{GFP}	5	133 ± 15	ns
	EC^{WT}	3	34 ± 2		EC^{WT}	4	118 ± 12	

Data represent mean ± s.e.m. SD: standard diet; WD: western-type diet; ALT: alanine transaminase; AST; aspartate transaminase; ns: not significant by Student's *t*-test.

Supplementary Note S2. *The code text used for RNAseq analyses*

```
## R code used for the analysis of RNA sequencing data

## Eskeatnaf Mulugeta

## Load necessary libraries

library(ggplot2)

library(edgeR)

library(RUVSeq)

library(ShortRead)

library(pheatmap)

library(gplots)

library(RColorBrewer)

library(DESeq2)

## Load target file

targets <- read.delim("targets.txt", stringsAsFactors = FALSE)

## Read files and check the data

d <- readDGE(targets, skip = 5, comment.char = "!")

d$samples  # Display sample information
```

```
head(d$counts)  # Show the first few rows of count data
tail(d$counts)  # Show the last few rows of count data
summary(d$counts)  # Provide a summary of count data
## Convert raw counts to counts per million (CPM)
cpm <- cpm(d)  # Raw counts in CPM
lcpm <- cpm(d, log = TRUE)  # Log-transformed CPM
## Calculate total counts and plot
# Count the number of genes with at least one read
sum(rowSums(d$counts) > 0)
# Compute total read counts per sample
totCounts <- colSums(d$counts)
totCounts  # Display total counts
# Bar plot of total read counts per library
barplot(totCounts,
    las = 2,
    col = c("red", "green")[factor(d$samples$Genotype_Diet)],
    ylab = "Total Counts",
    main = "Total Read Count per Library",
    cex.axis = 0.5,
    cex.names = 0.7)
# Remove low count and Filter
keep <- rowSums(cpm(d)>1) >= 3
d <- d[keep,]
dim(d)
boxplot(log(d$counts+0.01), col = c("red","Green")[factor(d$samples$Genotype_Diet)])
plotPCA(d$counts, col= c("red","Green","blue","black")[factor(d$samples$Genotype_Diet)])
## Remove low-count genes and filter data
# Keep genes with CPM > 1 in at least 3 samples
keep <- rowSums(cpm(d) > 1) >= 3
d <- d[keep, ]  # Filter dataset
dim(d)  # Check dimensions after filtering
# Boxplot of log-transformed counts
boxplot(log(d$counts + 0.01), col = c("red", "green")[factor(d$samples$Genotype_Diet)],
    main = "Boxplot of Log-transformed Counts")
# PCA plot of filtered counts
plotPCA(d$counts, col = c("red", "green", "blue", "black")[factor(d$samples$Genotype_Diet)],
```

```
    main = "PCA of Filtered Counts")
##Comparing the filtered and unfiltered data distribution
library(RColorBrewer)
nsamples <- ncol(d)
nsamples
col <- brewer.pal(nsamples, "Paired")
col
par(mfrow=c(1,2))
plot(density(lcpm[,1]), col=col[1], lwd=2, ylim=c(0,0.8), las=2,
   main="", xlab="")
title(main="A. Raw data", xlab="Log-cpm")
abline(v=0, lty=3)
for (i in 2:nsamples){
 den <- density(lcpm[,i])
 lines(den$x, den$y, col=col[i], lwd=2)
}
legend("topright", d$samples$Genotype_Diet, text.col=col, bty="n")
lcpm <- cpm(d, log=TRUE)
plot(density(lcpm[,1]), col=col[1], lwd=2, ylim=c(0,0.21), las=2,
   main="", xlab="")
title(main="B. Filtered data", xlab="Log-cpm")
abline(v=0, lty=3)
for (i in 2:nsamples){
  den <- density(lcpm[,i])
  lines(den$x, den$y, col=col[i], lwd=2)
}
legend("topright", d$samples$Genotype_Diet, text.col=col, bty="n")
# remove unwanted variations
#Please check the follwing manuscript for background behind this normalisation and the process of removing unwanted effects.
#Risso, D., J. Ngai, T. P. Speed, and S. Dudoit. 2014. Normalization of RNA-seq Data Using Factor Analysis of Control Genes or Samples." Nature Biotechnology.
x <- d$samples$Genotype
x
set <- newSeqExpressionSet(as.matrix(d$counts),phenoData = data.frame(x, row.names=colnames(d$counts)))
# plot Relative Log Expression (RLE): to visualize the differences between the distributions of read counts across samples.
```

plotRLE(set, outline=FALSE, ylim=c(-4, 4), col= c("red","Green")[factor(d$samples$Genotype_Diet)], main="RLE plot")

plotPCA(set, col= c("red","Green")[factor(d$samples$Genotype_Diet)], cex=1.2, main="PCA Colored with group")

Between lane normalization for sequencing depth and possibly other distributional differences between lanes.

set_2 <- betweenLaneNormalization(set, which="upper")

plotRLE(set_2, outline=FALSE, ylim=c(-4, 4), col= c("red","Green")[factor(d$samples$Genotype_Diet)], main="RLE plot")

plotPCA(set_2, col= c("red","Green")[factor(d$samples$Genotype_Diet)], cex=1.2, main="PCA Colored with group")

#Identifying Empirical control genes for RUVSeq (). For Empirical control genes refer to Risso, D., J. Ngai, T. P. Speed, and S. Dudoit. 2014. Normalization of RNA-seq Data Using Factor Analysis of Control Genes or Samples." Nature Biotechnology.

design <- model.matrix(~x , data=pData(set_2))

design

y <- DGEList(counts=counts(set_2), group=x)

y

y <- calcNormFactors(y, method="upperquartile")

y <- estimateGLMCommonDisp(y, design)

y <- estimateGLMTagwiseDisp(y, design)

fit <- glmFit(y, design)

lrt <- glmLRT(fit, coef=2)

top <- topTags(lrt, n=nrow(set))$table

top_deg <- subset(top,top$FDR<0.05)

top_deg_2 <- subset(top,top$PValue<0.05)

empirical <- rownames(set)[which(!(rownames(set) %in% rownames(top)[1:5000]))]

Remove unwanted variations

Refer to the following manuscript for background on this normalization approach and the process of removing unwanted effects:

Risso, D., J. Ngai, T. P. Speed, and S. Dudoit. 2014. "Normalization of RNA-seq Data Using Factor Analysis of Control Genes or Samples." Nature Biotechnology.

x <- d$samples$Genotype

x

Create a SeqExpressionSet object

set <- newSeqExpressionSet(as.matrix(d$counts),

phenoData = data.frame(x, row.names = colnames(d$counts)))

Plot Relative Log Expression (RLE) to visualize differences in read count distributions across samples

plotRLE(set, outline = FALSE, ylim = c(-4, 4), col = c("red", "green")[factor(d$samples$Genotype_Diet)],

main = "RLE Plot")

Principal Component Analysis (PCA) plot, colored by group

plotPCA(set, col = c("red", "green")[factor(d$samples$Genotype_Diet)], cex = 1.2, main = "PCA Colored by Group")

```
# Between-lane normalization to correct for sequencing depth and distributional differences

set_2 <- betweenLaneNormalization(set, which = "upper")

# Re-plot RLE after normalization

plotRLE(set_2, outline = FALSE, ylim = c(-4, 4), col = c("red", "green")[factor(d$samples$Genotype_Diet)],
        main = "RLE Plot After Normalization")

# Re-plot PCA after normalization

plotPCA(set_2, col = c("red", "green")[factor(d$samples$Genotype_Diet)], cex = 1.2, main = "PCA Colored by Group After Normalization")

## Identifying empirical control genes for RUVSeq.

## Refer to: Risso, D., J. Ngai, T. P. Speed, and S. Dudoit. 2014. "Normalization of RNA-seq Data Using Factor Analysis of Control Genes or Samples." Nature Biotechnology.

design <- model.matrix(~x, data = pData(set_2))

design

y <- DGEList(counts = counts(set_2), group = x)

y <- calcNormFactors(y, method = "upperquartile")  # Normalize library sizes

y <- estimateGLMCommonDisp(y, design)

y <- estimateGLMTagwiseDisp(y, design)

fit <- glmFit(y, design)

lrt <- glmLRT(fit, coef = 2)

# Extract top differentially expressed genes (DEGs)

top <- topTags(lrt, n = nrow(set))$table

# Filter for significant DEGs

top_deg <- subset(top, top$FDR < 0.05)  # Using False Discovery Rate (FDR)

top_deg_2 <- subset(top, top$PValue < 0.05)  # Using p-value

# Identify empirical control genes (genes not in the top 5000 differentially expressed genes)

empirical <- rownames(set)[which(!(rownames(set) %in% rownames(top)[1:5000]))]

## Remove Unwanted Variation (RUV) using control genes and plot RLE & PCA

set_3 <- RUVg(set_2, empirical, k=1)

pData(set_3)

plotRLE(set_3, outline=FALSE, ylim=c(-4, 4), col= c("red","Green")[factor(d$samples$Genotype_Diet)], main="RLE plot")

plotPCA(set_3, col= c("red","Green" )[factor(d$samples$Genotype_Diet)], cex=1.2, main="PCA Colored with group")

## Extract the normalized data

set3_norm<- normCounts(set_3)

colnames(set3_norm) <-d$samples$info3

head(set3_norm)

# Calculate Sample Distance: Euclidean Distance and Plot Heatmap
```

```
sampleDists<- dist( t( log(set3_norm+1,2) ) )
sampleDists
sampleDistMatrix <- as.matrix( sampleDists )
rownames(sampleDistMatrix) <- paste( d$samples$Genotype_Diet,d$samples$Biomics_ID,sep="-"  )
colnames(sampleDistMatrix) <- paste(d$samples$Genotype_Diet,d$samples$Biomics_ID,sep="-"  )
pheatmap(sampleDistMatrix,
        clustering_distance_rows=sampleDists,
        clustering_distance_cols=sampleDists,
        clustering_method="ward.D2", fontsize = 9,
        main= "Clustered Heatmap of Sample Distances")
## Identify Differentially Expressed Genes (DEGs)
design <- model.matrix(~x + W_1, data=pData(set_3))
design
y <- DGEList(counts=counts(set_2), group=x)
y <- calcNormFactors(y, method="TMM")
y
lcpm_y<- cpm(y, normalized.lib.sizes=TRUE, log=FALSE)
y <- estimateGLMCommonDisp(y, design)
y
y <- estimateGLMTagwiseDisp(y, design)
y
fit <- glmFit(y, design)
lrt <- glmLRT(fit, coef=2)
topTags(lrt)
## Generate Volcano Plot
plot(top$logFC, -log10(top$PValue), pch=1, cex=.1, ylab="-log10(p-value)", xlab="logFC", col=as.numeric(rownames(top) %in% de)+1, main="Volcano plot", xlim=c(-12,12))
abline(v=c(-1, 1), col=4 )
abline(v=c(-2, 2), col=3 )
## Summarizing results
all_sig.DGEs <- rownames(top)[top$FDR < 0.05 ]  ### Total differentially expressed genes (DEGs) without cutoff
sig.DGEs <- rownames(top)[top$FDR < 0.05 & abs(top$logFC) > 0.25]  ##  Differentially expressed genes (DEGs) with 0.25 log fold change (abs, absolute value of 0.25)
sig.upDGEs <- rownames(top)[top$FDR < 0.05 & top$logFC > 0.25] ## Up-regulated DEGs with 0.25 log fold change
sig.downDGEs <- rownames(top)[top$FDR < 0.05 & top$logFC < -0.25] ## Down-regulated DEGs with 0.25 log fold change
```

D. References in supplement

1 Birkhoff, J. C., Huylebroeck, D. & Conidi, A. ZEB2, the Mowat-Wilson Syndrome Transcription Factor: Confirmations, Novel Functions, and Continuing Surprises. *Genes (Basel)* **12**, doi:10.3390/genes12071037 (2021).

2 Guilliams, M. *et al.* Spatial proteogenomics reveals distinct and evolutionarily conserved hepatic macrophage niches. *Cell* **185**, 379-396 e338, doi:10.1016/j.cell.2021.12.018 (2022).
